# Stereochemical Control of Cu(II) and Zn(II) Binding in Clavanin C Peptidomimetics

**DOI:** 10.1021/acs.inorgchem.6c02172

**Published:** 2026-06-25

**Authors:** Jakub Gawłowski, Mariusz Dziadas, Aleksandra Mikołajczyk-Tarnawa, Arian Kola, Daniela Valensin, Agnieszka Matera-Witkiewicz, Magdalena Rowińska-Żyrek

**Affiliations:** † Faculty of Chemistry, 49572 University of Wrocław, F. Joliot-Curie 14, 50-383 Wrocław, Poland; ‡ Screening Laboratory of Biological Activity Tests and Collection of Biological Material, Faculty of Pharmacy, 49550 Wroclaw Medical University, Borowska 211A, 50-556 Wrocław, Poland; § Department of Biotechnology, Chemistry and Pharmacy, University of Siena, Via A. Moro 2, 53100 Siena, Italy; ∥ CIRMMP, Via Luigi Sacconi 6, 50019 Sesto Fiorentino, Italy

## Abstract

We redesign the histidine-rich antimicrobial peptide clavanin C through stereochemical inversion to generate proteolytically stable metallopeptidomimetics while preserving metal-binding functionality and antimicrobial activity. Two analogues were prepared: an all-d amino acid peptide and a *retro-inverso* variant. Remarkably, despite complete sequence reversal and inversion of chirality, both mimetics retain Cu^2+^ coordination through the N-terminal ATCUN motif, forming characteristic square-planar 4N complexes. Zn^2+^ binding is likewise preserved and involves a cluster of histidine residues located on one face of the amphipathic α-helix. This distinction reveals two complementary design principles: sequence-encoded metal binding in the case of Cu^2+^ versus topology-driven coordination for Zn^2+^. Both stereochemical designs dramatically increase resistance to proteolytic degradation while maintaining the helical fold under membrane-mimicking conditions. In antimicrobial assays, the *retro-inverso* analogue and its metal complexes display particularly strong activity against methicillin-resistant *Staphylococcus aureus* (MRSA), with minimum inhibitory concentrations as low as 8 μg/mL and no detectable cytotoxicity toward mammalian cells. Taken together, our data show that both d-amino acid substitution and retro-inverso design can stabilize clavanin C while retaining its metal-binding ability and antimicrobial activity, providing a general strategy for the development of proteolytically stable antimicrobial metallopeptidomimetics.

## Graphical abstract

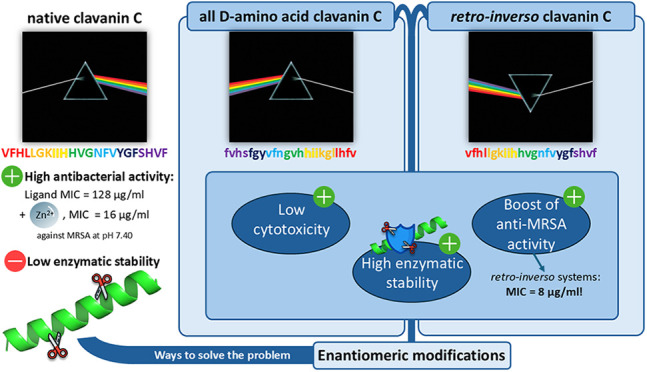

## Introduction

The rapid spread of multidrug-resistant bacteria represents a major global health challenge, with projections estimating up to 40 million deaths annually by 2050.^1^ This escalating threat has intensified the search for alternative therapeutic strategies, among which antimicrobial peptides (AMPs) have emerged as promising candidates due to their broad-spectrum activity and central role in innate immunity.^2^

Marine organisms constitute a rich source of structurally diverse AMPs, including piscidins, clavanins, arenicins and aurelin. Among these, clavanins represent a particularly intriguing class of histidine-rich, cationic peptides isolated from the hemocytes of the tunicate *Styela clava*.^1,2^ This family comprises five closely related peptides (A–E), each consisting of 23 amino acid residues and adopting an α-helical conformation under membrane mimicking environment (Figure 1).^3^

**Figure 1:**
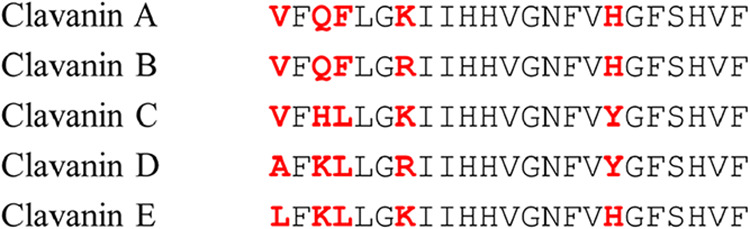
Amino acid sequences of clavanin A–E. APD ID numbers:AP00276–AP00280. The differences between peptides are highlighted.

Clavanins exhibit broad antimicrobial activity against both Gram-positive and Gram-negative bacteria as well as fungi, particularly under mildly acidic conditions (pH 5.50).^1,4^ Beyond their antimicrobial effects, clavanins display additional biological activities, such as the pro-regenerative effects reported for clavanin A.^7^ Among them, clavanin C demonstrates particularly strong activity against clinically relevant pathogens, including *Staphylococcus aureus*, *Enterococcus faecalis* and *Candida albicans*. Importantly, the antimicrobial activity of clavanins can be modulated by coordination with divalent metal ions, particularly Zn^2+^ and Cu^2+^.^5^ In recent years, increasing attention has been devoted to the role of metal ions in modulating the activity of antimicrobial peptides. Metal coordination can influence peptide structure, membrane interactions, and antimicrobial potency, making metallopeptides an emerging strategy in antimicrobial design.^6,7^ Beyond their role in nutritional immunity,^11^ metal ions can directly modulate antimicrobial activity when coordinated by peptides by altering their net charge or secondary structure, or by enabling additional mechanisms of action,^8,9^ making metal coordination an increasingly attractive strategy in AMP design. However, how these metal-binding properties respond to extensive stereochemical modifications of the peptide backbone remains largely unexplored. Despite their potent antimicrobial properties, many AMPs suffer from rapid proteolytic degradation in biological environments, which significantly limits their therapeutic potential. Both host- and pathogen-derived proteases can rapidly inactivate peptide antibiotics, making the development of proteolytically stable analogues a major challenge in AMP-based drug design.^14^ To address it, numerous strategies have been developed to enhance peptide resistance to proteolytic degradation. These include N- and C-terminal modifications, cyclization, backbone alterations, incorporation of noncanonical amino acids, and various forms of conjugation.^10–14^ Among these alterations, stereochemical modification represents one of the most effective approaches. Substitution of l-amino acids with their d-counterparts significantly reduces susceptibility to enzymatic degradation, as proteases typically recognize only the natural L-configuration. Importantly, such modifications often preserve the amphipathic and α-helical character of antimicrobial peptides, allowing retention of biological activity.^15^

Another promising approach involves the use of *retro-inverso* (RI) peptides,^16^ in which both sequence direction and chirality are inverted (Figure 2). This modification results in peptidomimetics that maintain the spatial arrangement of side chains – these are superimposable on those of the parent L-peptide – but feature inverted amide bonds and reversed N- and C-terminal groups. This design preserves the spatial arrangement of side chains while fundamentally altering the peptide backbone, often maintaining biological function while further enhancing resistance to proteolysis.^17^

**Figure 2:**
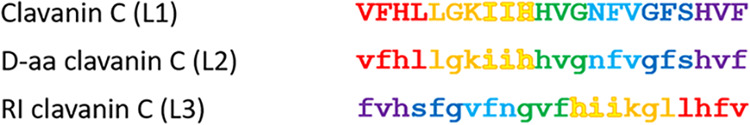
Amino acid sequences of clavanin C (L1) and its analogues: D-clavanin C (L2) and RI-clavanin C (L3). N-terminal part (of native clavanin C) is in red, C-terminal part- in violet. l-amino acids are in capitals, d-amino acids are in low-case.

Despite the growing interest in *retro-inverso* antimicrobial peptides, very little is known about how such stereochemical inversion affects metal-binding properties and metallopeptide activity. In particular, it is unclear whether well-defined metal-binding motifs and coordination geometries can be preserved upon such extensive stereochemical perturbations of the peptide backbone. Only a limited number of studies have addressed the coordination chemistry of D- or *retro-inverso* antimicrobial peptides, including investigations on MUC7-derived peptides, which demonstrated that stereochemical inversion can alter metal-binding thermodynamics and, consequently, biological activity.^18,19^

Encouraged by our previous results on the complexes of native clavanin C with Cu^2+^ and Zn^2+^,^8^ in this study, we investigate how stereochemical inversion through d-amino acid substitution and *retro-inverso* design affects metal coordination, structural properties, and antimicrobial activity of clavanin C. Given the presence of a defined N-terminal metal-binding motif, this system provides a unique opportunity to assess the robustness of sequence-encoded coordination under extreme stereochemical perturbation. To this end, we designed two proteolytically stable analogues: an all-D variant (L2) and a *retro-inverso* analogue (L3), and compared their metal-binding properties and biological activity with those of the native peptide (L1). Building on our previous studies of Cu^2+^ and Zn^2+^ complexes of clavanin C, we aim to establish how stereochemical redesign influences metal binding, structure, and function, with the goal of developing stable metallopeptidomimetics that retain high antimicrobial activity while minimizing cytotoxicity.

## Experimental Methods

### Materials

All peptides: clavanin C (VFHLLGKIIHHVGNFVYGFSHVF) and their two mimetics – all d amino acid peptide (vfhllgkiihhvgnfvygfshvf) and *retro-inverso* peptide (fvhsfgyvfngvhhiikgllhfv) were synthesized commercially by KareBay Biochem (certified purity: 98%) and were used as received. The concentrations of Cu^2+^ and Zn^2+^ perchlorate hexahydrate (Sigma-Aldrich) solutions were determined using inductively coupled plasma optical emission spectrometry (ICP-OES). Carbonate-free 0.1 M NaOH solution (Sigma-Aldrich) was standardized with potassium hydrogen phthalate (Sigma-Aldrich) and used as a titrant in the potentiometric experiments. The ionic strength (I) was 40 mM SDS (Sigma-Aldrich) and perchloric acid (Supelco) was used to adjust the pH of the samples. All samples were prepared by using double-distilled water.

### Electrospray Ionization-Mass Spectrometry (ESI-MS)

Mass spectra of investigated systems (ligands and their complexes with Cu^2+^ and Zn^2+^ ions) were acquired on a Bruker Compact QTOF (Bruker Daltonik, Germany) equipped with an electrospray ionization source with an ion funnel. All spectra were recorded in positive ion modes, within a mass-to-charge ratio (*m*/*z*) range of 150–3000. The experimental conditions were as follows: dry nitrogen gas, *T* = 180 °C, a capillary voltage of 4500 V, ion energy of 5 eV. Solutions of ligands and their metal complexes ([L] = 0.1 mM, molar ratio M: L = 1:1) were prepared by dissolving in a 50:50 (volume ratio) mixture of water and MeOH (Supelco). The instrument was calibrated externally with a Low Concentration Tuning Mix ESI-ToF (Agilent Technologies, Santa Clara, CA). The samples were infused at a flow rate of 3 μL min^–1^. Obtained spectra were analyzed using the Bruker Compass DataAnalysis 6.1 program.

### Potentiometry

The stability constants for all ligand and their complexes with Cu^2+^ and Zn^2+^ ions were calculated based on titration curves in the pH range 2.0–12.0 at 25 °C in a total volume of 2.6 mL. Potentiometric measurements were carried out in 4 mM HCLO_4_ solution with ionic strength *I* = 40 mM SDS for all systems. A Metrohm Titrando 905 titrator equipped with a Mettler Toledo InLab Semi-Micro combined pH electrode. The glass cell, maintained at a constant temperature, was fitted with a magnetic stirring device, a microburet for precise liquid dispensing, and a tube system for argon flow. Solutions were titrated with 0.1 M carbonate-free NaOH. Experiments were proceeded by electrode calibration involving 4 mM HCLO_4_ titration with 0.1 M NaOH using a total volume of 3 mL. Determination of concentration and purity level was possible through using the Gran method.^20^ The ligand concentration was 0.4 mM and the M: L molar ratio was 0.8:1. Calculations of constant stability were performed in HYPERQUAD 2008.^21^ The standard deviations were computed by using HYPERQUAD 2008 and referenced to random errors only. The hydrolysis constants of Cu^2+^ and Zn^2+^ were taken from literature and used in these calculations.^22^ The speciation and competition diagrams were computed using the HYSS program and visualized in the Origin 2024 program.^23,24^

### Spectroscopic Studies

The Absorption spectra were recorded on a Jasco V-750 spectrophotometer, while circular dichroism (CD) spectra were acquired on a Jasco J-1500 CD spectropolarimeter; in both cases, a quartz cuvette with an optical path length of 1 cm was used. For far-UV CD experiment (wavelength range 180–250 nm), a quartz cuvette with an optical path length of 0.2 mm was used. UV–vis and CD experiments were conducted in a pH range of 3.0–11.0 at 25 °C. The pH values of samples were adjusted by adding NaOH and HClO_4_ solutions. Concentration of samples were similar to those used in the potentiometric titration; the metal-to-ligand molar ratio was 0.8 to 1.0.

NMR spectra were obtained on a Bruker Avance III 600 MHz spectrometer, operating at a magnetic field strength of 14.1 T, equipped with a 5 mm BBI (Broad Band Inverse) probe. The temperature was set and maintained at 298 K with an accuracy of ± 0.1 K. The residual water signal was suppressed using excitation sculpting, employing a 2 ms selective square pulse on the water resonance. All samples were prepared in a mixture of 90% H_2_O and 10% D_2_O, containing 40 mM SDS-*d*_26_. TMSP (3-(Trimethylsilyl)­propionic-2,2,3,3-d_4_) acid sodium salt (2 mM, Sigma-Aldrich) was used as an internal reference standard. A 20 mM phosphate buffer was used to maintain pH 7.40. Proton resonance assignment was achieved using 2D ^1^H–^1^H total correlation spectroscopy (TOCSY) and nuclear Overhauser effect spectroscopy (NOESY) experiments, conducted with standard pulse sequences. Data processing and analysis were completed using the Bruker TOPSPIN 3.6.5 software. Complexes were prepared by adding appropriate equivalents of Zn­(ClO_4_)_2_ to the 0.5 mM ligand’s solution.

### Peptide Digestion

Three synthetic peptides were used in this study: native clavanin C (L1), its d-amino acid variant (L2), and retro-inverso analogue (L3). All peptides were synthesized via solid-phase peptide synthesis (SPPS) using Fmoc chemistry and purified to >98% by HPLC. Peptides were dissolved in a methanol–water mixture (volume ratio 10:90) to a final concentration of 1 mg/mL. Trypsin (Sigma-Aldrich) was added at an enzyme-to-substrate ratio of 1:50 (w/w). Tween 20 was included to reduce unwanted interactions between the peptide and enzyme, including nonspecific binding and aggregation, which could hinder digestion. Digestion was performed at 37 °C for 1 h. Reactions were quenched by acidification with TFA (final concentration 0.1%).

### HPLC Analysis

Synthetic peptide standards (undigested) and digestion products were analyzed using liquid chromatography coupled with UV detector. Chromatographic separation was performed on a Kinetex, 100 mm × 3 mm, 2.6 μm, C18 RP column (Phenomenex) using a gradient of acetonitrile and water (both containing 0.1% formic acid). The gradient was programmed as follows: 5% B from 0.0 to 0.5 min; linear increase to 100% B by 11.0 min; held at 100% B from 11.0 to 12.0 min; returned to 5% B at 12.1 min and held until 17.0 min for re-equilibration. The flow rate was 0.35 mL/min and the column temperature was maintained at 30 °C.

### Antimicrobial Activity Assay of Peptide and Peptide–Metal Ion Complex System

Five reference strains obtained from ATCC collection [*Pseudomonas aeruginosa* (ATCC 27853), *Escherichia coli* (ATCC 25922), *Staphylococcus aureus* MRSA (ATCC 43300), *Enterococcus faecalis* (ATCC 29212), and *Candida albicans* (ATCC 10231)] were used for antimicrobial activity testing. The antimicrobial effect of the analyzed peptides/complexes was evaluated using a broth microdilution method with spectrophotometric measurement (λ = 580 nm at starting point and after 24 h) according to the ISO standard 20776-1:2019, ISO standard 16256:2021 and modified Richard’s method.^25–28^ Stock peptide solutions were prepared in deionized sterile water at a 4-fold higher concentration than the highest tested concentration. Serial dilutions of the ligand/complex solution were made on 96-well microplates in the range of 0.5–256 μg/mL (pH = 7.4); concentrations correspond to the total peptide concentration in the metal-peptide systems. Tryptone Soya Agar plates were inoculated with microbial strains from glycerol stocks. After 24 h of incubation at 37 °C (for bacteria) or 25 °C (for fungus), an appropriate bacterial and fungal cell density was prepared using a densitometer [final inoculum (5 × 10^5^ CFU/mL for bacteria and 0.5–2.5 × 10^5^ CFU/mL for fungus) in Tryptic Soy Broth (TSB)]. Positive (TSB + strain) and negative controls (TSB) were also included in the test. Spectrophotometric solubility control of each peptide and the peptide–metal ion system was also performed. For each strain, the validation process was performed using the following antibacterial/antifungal agents: levofloxacin, gentamicin, and amphotericin B according to the EUCAST examination. The minimum inhibitory concentration (MIC) was determined as the lowest concentration of an antimicrobial agent that decreased the measured microbial growth to below approximately 50% relative to the positive control. The obtained MIC values for reference antibiotics were as follows: *E. coli* (ATCC 25922): gentamicin 4 μg/mL; *E. faecalis* (ATCC 29212): levofloxacin 4 μg/mL; *P. aeruginosa* (ATCC 27853): levofloxacin 1 μg/mL; *S. aureus* MRSA (ATCC 43300): levofloxacin 1 μg/mL; *C. albicans* (ATCC 10231): amphotericin B 1 μg/mL. Microplates were incubated at 37 ± 1 °C or 25 ± 1 °C for 24 h on the shaker (500 rpm). Subsequently, the spectrophotometric measurement was performed at 580 nm, and then 50 μL aliquots of 1% (m/v) 2,3,5-triphenyltetrazolium chloride (TTC) solution were added into each well. TTC is a chemical indicator that is converted into red formazan crystals in living microbial cells. The minimum bactericidal concentration (MBC) or the minimum fungicidal concentration (MFC) was defined as the lowest concentration required to kill a particular microbial strain, determined by visual analysis after 24 h incubation with TTC (remained colorless, indicating no formazan formation).

### Neutral Red Cytotoxicity Assay

Cytotoxicity of the peptides and peptide-metal ion complexes was evaluated using a neutral red (NR) uptake assay in L929 fibroblast cells. The assay was performed according to a standard protocol.^29^ L929 cells were seeded into 96-well plates at a density of 1 × 10^4^ cells per well and allowed to attach for 24 h. The tested compounds (clavanin C and its D-aa and RI analogs, as well as their complexes with Cu^2+^ and Zn^2+^ ions) were added at final concentrations of 1, 10, 20, and 30 μM. The selected concentration range corresponded to previously determined antimicrobial activity (MIC 8–64 μg/mL; approximately 3–24 μM). Each concentration was tested in triplicate. Additionally, precipitation controls for the compounds, a negative control (untreated cells cultured in medium only), and a positive control (cells treated with 1 μM staurosporine) were included.

After 24, 48, and 72 h of incubation with the compounds, the NR uptake assay was performed. Briefly, the culture medium was removed and replaced with 100 μL of neutral red solution (40 μg/mL), followed by incubation for 2 h at 37 °C. The dye solution was then discarded, and the cells were washed with PBS. Subsequently, 100 μL of extraction solution (49% ethanol, 50% H_2_O, and 0.1% glacial acetic acid) was added, and the plates were shaken for 30 min to solubilize the incorporated dye. Absorbance was measured at 540 nm.

## Results

### Stoichiometry

Electrospray ionization mass spectrometry confirmed the formation of Cu^2+^ and Zn^2+^ complexes with clavanin C and its stereochemical analogues (D-aa clavanin C and *retro-inverso* clavanin C, L1–L3). Comparison of experimental and simulated isotopic patterns revealed exclusively mononuclear species with a 1:1 metal-to-ligand stoichiometry under the investigated conditions (Figure S1), indicating a well-defined and consistent coordination regime across all systems.

### Ligand Deprotonation

Six protonation equilibria were identified for all three ligands (L1–L3), corresponding to four histidine imidazole groups, the N-terminal amine, and the tyrosine phenolic group (Table 1). The obtained p*K*_a_ values are consistent with literature data,^30–34^ and no C-terminal deprotonation was observed within the investigated pH range. Species distribution diagrams are shown in Figure S2.

**Table 1:** Deprotonation Constants (p*K*_a_) for L1, L2, and L3 Peptides and Stability Constants (log β) for Their Complexes with Cu^2+^ and Zn^2+^ Ions in Solution of 4 mM HClO_4_ with I = 40 mM SDS at 25 °C. C_L_ = 0.4 mM^a^

species	vlavanin C (L1) VFHLLGKIIHHVGNFVYGFSHVF			D-clavanin C (L2) vfhllgkiihhvgnfvygfshvf			RI-clavanin C (L3) fvhsfgyvfngvhhiikgllhfv		
	logβ	p*K*_a_		logβ	p*K*_a_		logβ	p*K*_a_	
[H_6_L]^6+^	48.17(2)	5.36	–N_im_ (His)	48.38(2)	5.37	–N_im_ (His)	48.88(5)	5.72	–N_im_ (His)
[H_5_L]^5+^	42.81(2)	7.10	–N_im_ (His)	43.01(2)	7.11	–N_im_ (His)	43.16(5)	7.24	–N_im_ (His)
[H_4_L]^4+^	35.71(2)	7.60	–N_im_ (His)	35.90(2)	7.65	–N_im_ (His)	35.92(5)	7.77	–N_im_ (His)
[H_3_L]^3+^	28.11(2)	8.17	–N_im_ (His)	28.25(2)	8.20	–N_im_ (His)	28.15(5)	8.41	–N_im_ (His)
[H_2_L]^2+^	19.94(2)	8.95	–NH_2_	20.05(1)	8.94	–NH_2_	19.74(4)	8.86	–NH_2_
[HL] ^+^	10.99(1)	10.99	–OH (Tyr)	11.11(1)	11.11	–OH (Tyr)	10.88(4)	10.88	–OH (Tyr)
*Zn^2+^ complexes*									
[ZnH_4_L]^6+^	39.70(8)			39.96(8)			39.70 (8)		
[ZnH_3_L ]^5+^									
[ZnH_2_L]^4+^	25.07(4)			25.43(5)			25.06(4)		
[ZnHL]^3+^									
[ZnL]^2+^	8.14(6)			8.60(6)			8.22(5)		
[ZnH_‑1_L]^+^									
[ZnH_‑2_L]	–11.65(8)			–10.99(7)			–11.47(6)		
*Cu^2+^ complexes*									
[CuH_5_L]^7+^	46.33(10)						46.99(7)		
[CuH_4_L]^6+^	41.39(3)	4.94		41.41(3)			41.69(5)	5.45	
[CuH_3_L]^5+^									
[CuH_2_L]^4+^	30.28(2)			29.92(2)			30.35(2)		
[CuHL]^3+^	23.82(3)	6.46	–N_im_ (His)	23.67(3)	6.28	–N_im_ (His)	23.52(5)	6.83	–N_im_ (His)
[CuL]^2+^	16.45(3)	7.37	–N_im_ (His)	16.24(3)	7.43	–N_im_ (His)	15.40(4)	8.12	–N_im_ (His)
[CuH_–1_L]^+^	7.86(4)	8.59	–N_im_ (His)	7.87(4)	8.37	–N_im_ (His)	6.57(4)	8.83	–N_im_ (His)
[CuH_–2_L]	–2.92(4)	10.78	–OH (Tyr)	–3.14(6)	11.01	–OH (Tyr)	–4.08(5)	10.65	–OH (Tyr)

a The standard deviations are reported in parentheses as uncertainties on the last significant figure.

For native clavanin C (L1), the first four deprotonation steps are attributed to histidine imidazole nitrogens, followed by deprotonation of the N-terminal amine and the tyrosine side chain. The overall protonation pattern is in excellent agreement with previous reports.^5^

Importantly, the p*K*_a_ values determined for the d-amino acid and *retro-inverso* analogues (L2 and L3) show only minor variations (L2:5.37, 7.11, 7.65, 8.20, 8.94, 11.11 and L3:5.72, 7.24, 7.77, 8.41, 8.86, 10.88, respectively), while preserving the order of deprotonation of all functional groups. This indicates that stereochemical inversion and sequence reversal do not significantly perturb the intrinsic acid–base properties of the coordinating residues. Consequently, the protonation state of key donor groups, including histidine imidazoles and the N-terminal amine, remains largely conserved across all systems.

### Zn^2+^ Complexes

For the Zn^2+^-L1 system, four protonation-dependent species are formed across the investigated pH range (Table 1, Figure S3A, in perfect agreement with our previously published data^5^), with the physiologically relevant region centered around pH ∼7.4. Under these conditions, Zn^2+^ coordination is dominated by histidine imidazole donors. Since Zn^2+^ complexes are spectroscopically silent in the d–d region, DFT calculations allow to gain insight into the coordination sphere, revealing that at pH 7.40 Zn^2+^ is coordinated by three imidazole nitrogen atoms {3N_im_}: His10, His11, and His21.^5^

For the Zn^2+^-L2 complex, the speciation pattern closely resembles that observed for the native peptide (L1) (Table 1, Figure S3B), with similar stability constants across the entire pH range. The first species observed at acidic pH, [ZnH_4_L]^6+^, reaches its maximum abundance around pH 6.8. By analogy to the native peptide and considering the very similar stability constants, this form most likely involves only one histidine imidazole in Zn^2+^ binding. Upon further deprotonation, the [ZnH_2_L]^4+^ species becomes dominant in the physiological pH range, which is consistent with the involvement of three histidine imidazole donors in metal coordination, as proposed for the native system. The subsequent species, [ZnL]^2+^ and [ZnH_–2_L], are formed at higher pH and most likely arise from deprotonation of noncoordinating groups and additionally bound water molecules, without major changes in the Zn^2+^ coordination mode. Overall, the close similarity of the stability constants strongly suggests that d-amino acid substitution does not significantly alter the Zn^2+^ binding mode. This strongly suggests that the coordination mode is preserved upon d-amino acid substitution. By analogy to L1, Zn^2+^ binding at acidic pH likely involves a single histidine imidazole, followed by progressive recruitment of additional imidazole donors with increasing pH. Around physiological pH, the dominant species corresponds to a {3N_im_} coordination mode, in which three histidine residues participate in metal binding, while the N-terminal amine remains noncoordinating.

The similarity of stability constants for the Zn^2+^–L3 system compared to L1 and L2 suggests that Zn^2+^ coordination in the *retro-inverso* analogue also involves three histidine imidazole donors. To identify the residues participating in metal binding, NMR spectroscopy was employed. At pH 7.40, addition of Zn^2+^ induced clear chemical shift perturbations for His3, His13, and His14, while the signals corresponding to His21 remained unaffected (Figure 3).

**Figure 3:**
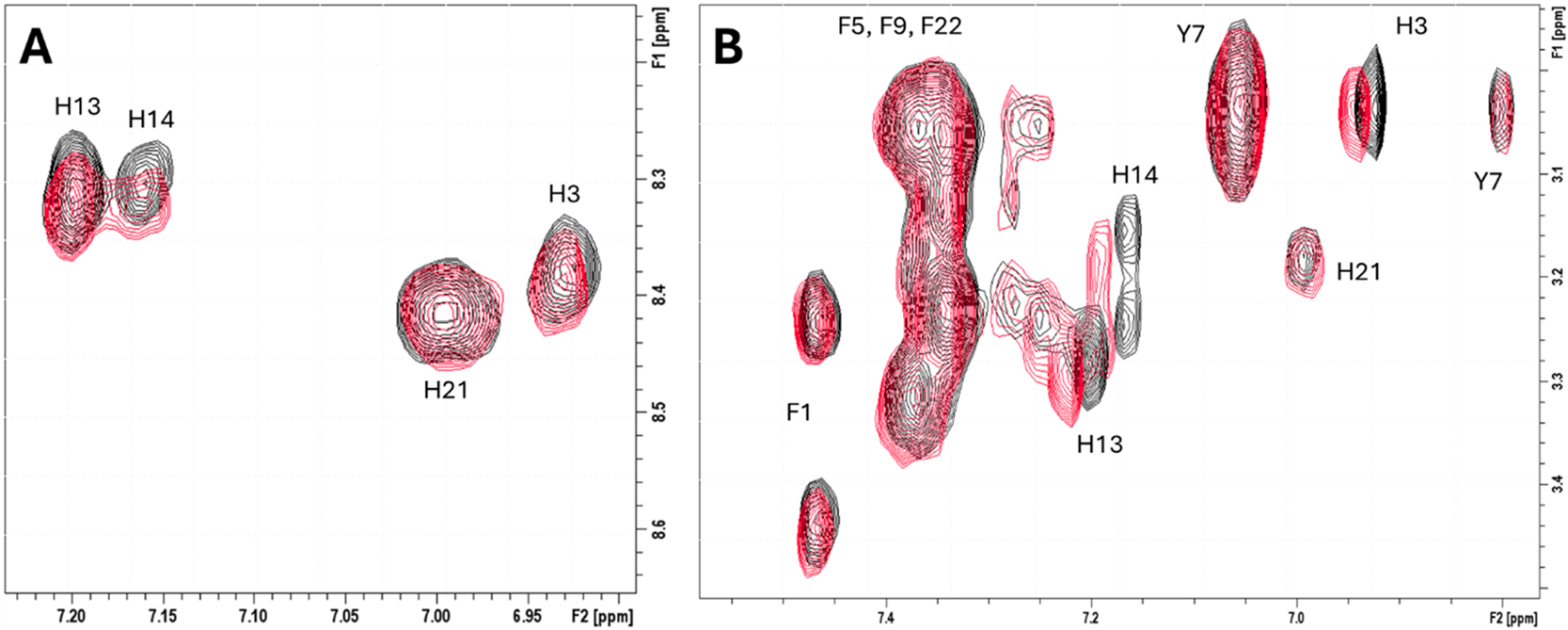
Aromatic regions of 2D NMR spectra: (A) Hδ−Hε cross-peaks ^1^H–^1^H TOCSY and (B) ^1^H–^1^H NOESY Hδ−Hβ cross-peaks of retro-inverso clavanin C in solution of 40 mM SDS-*d*_26_ at 25 °C. C_L_ = 0.5 mM, pH 7.40, in absence (black) and in the presence of 0.8 Zn^2+^ eqs (red).

The selective involvement of these residues can be rationalized in terms of the peptide’s α-helical topology. Under membrane-mimicking conditions, clavanin C adopts an amphipathic helix, in which His13 and His14 form a proximal pair positioned on the same face of the helix, creating a favorable binding site. Although residues in i and i+1 positions are typically oriented differently due to the helical rotation, the conformational flexibility of imidazole side chains allows both residues to adopt rotamers compatible with metal coordination. His3, located near the N-terminus, contributes to Zn^2+^ binding due to increased conformational flexibility in this region, which is less structurally constrained than the helical core. In contrast, His21 is positioned on the opposite face of the helix relative to the binding site and remains spatially separated from the coordinating residues. As a result, it cannot participate in metal binding without significant disruption of the helical structure, consistent with the absence of chemical shift changes in the NMR spectra. Taken together, these results support a coordination model in which Zn^2+^ is bound by three histidine residues (His3, His13, and His14) clustered along one face of the helix (Figure 4). Notably, although different histidine residues are involved compared to the native peptide, their spatial arrangement within the α-helical framework is preserved. This indicates that Zn^2+^ binding is governed primarily by the topology of the peptide rather than by the specific sequence identity of the coordinating residues.

**Figure 4:**
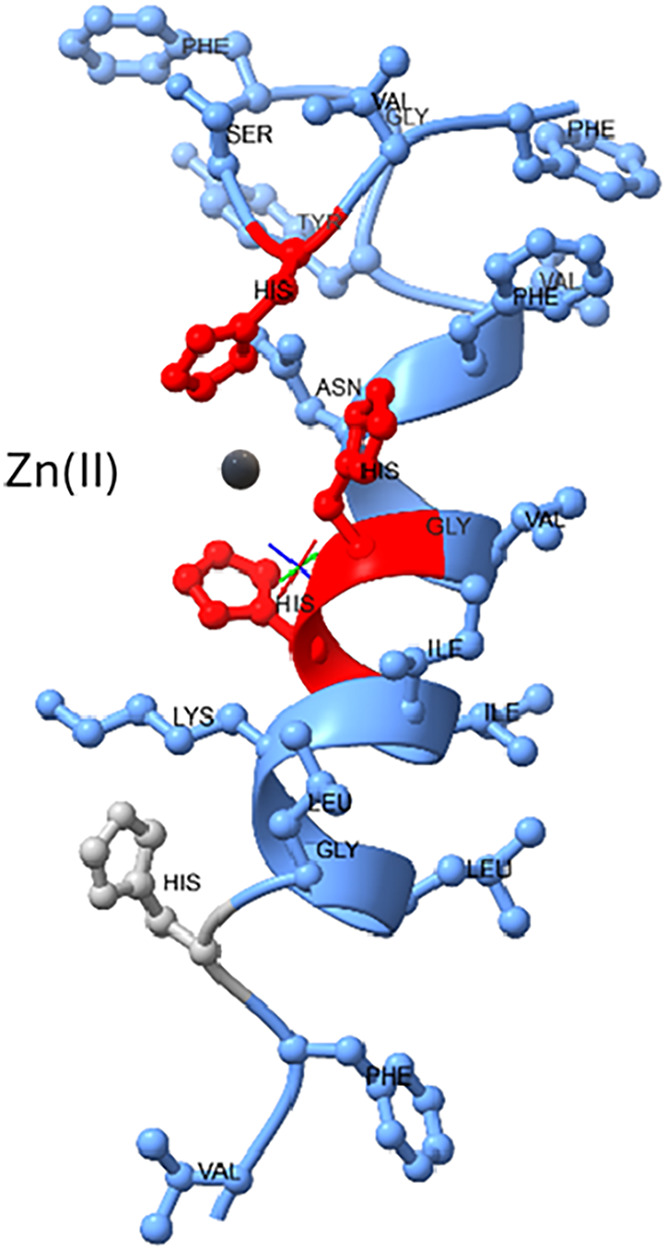
Representative illustration of Zn^2+^ coordination in complex with retro-inverso clavanin C based on NMR data.

### Cu^2+^ Complexes

All three ligands contain a very specific ATCUN motif (Xaa–Yaa–His), which is capable of forming very stable square-planar complexes with Cu^2+^ and Ni^2+^ ions.^35–37^

For the native peptide (L1), Cu^2+^ binding is initiated around pH ∼6.0 with the formation of a [NH_2_, 2N^–^, N_im_] coordination environment, as evidenced by a characteristic d–d band at 517 nm and CD signals consistent with square-planar geometry (Figure S6A). At higher pH, subsequent deprotonation steps correspond to noncoordinating residues and do not alter the core 4N coordination mode, indicating the formation of a stable and well-defined ATCUN-type complex. These observations are in perfect agreement with our previous studies on clavanin C.^5^

In case of Cu^2+^-L2, copper­(II) coordination starts at around pH 6.00, where the predominant complex species is [CuH_2_L]^4+^ (Table 1, Figure S4B). The presence of a d-d transition band in the UV–vis spectrum at pH 6.00, with the highest molar extinction coefficient at 518 nm, confirms that Cu^2+^ is coordinated by four nitrogen atoms in an ATCUN-type coordination mode (Figure S5B).^8^

Importantly, despite complete inversion of amino acid chirality, the Cu^2+^ coordination behavior of L2 closely resembles that of the native peptide. The CD spectra show the expected mirror-image pattern relative to L1, reflecting the opposite handedness of the ligand, while the spectroscopic signatures of the square-planar [NH_2_, 2N^–^, Nim] binding mode remain preserved (two bands with a positive Cotton effect at λ = 558 nm and a negative one at 485 nm (Figure S6B)^5,35,38^ ). This demonstrates that inversion of chirality alone does not disrupt ATCUN-type Cu^2+^ coordination.

Further deprotonation steps correspond to noncoordinating histidine residues (His10, His11, and His21 (Figure 2), with p*K*_a_ values: 6.28, 7.43 and 8.37, respectively) and, at higher pH, to the tyrosine side chain, without altering the core 4N coordination mode.

For the *retro-inverso* Cu^2+^–L3 system, copper­(II) coordination starts around pH 6.0, with [CuH_2_L]^4+^ as the predominant species (Figure S4C). The presence of a d–d transition band at 519 nm confirms the formation of a square-planar 4N coordination environment characteristic of ATCUN-type binding (Figure S5C). The overall pH-dependent spectroscopic behavior closely parallels that observed for both the native peptide (L1) and its d-amino acid analogue (L2).

Remarkably, despite complete sequence reversal combined with full inversion of backbone chirality, the Cu^2+^ coordination mode remains unchanged. The spectroscopic signatures unequivocally demonstrate preservation of the canonical {NH_2_, 2N^–^, Nim} binding environment, indicating that the ATCUN motif retains its structural and functional integrity under extreme stereochemical perturbation.

Subsequent deprotonation steps, corresponding to noncoordinating histidine residues ([CuHL]^3+^, [CuL]^2+^ and [CuH_–1_L]^+^, with p*K*_a_ values = 6.83, 8.12 and 8.83, respectively (Table 1, Figure S4C)) and, at higher pH, to the tyrosine side chain, do not affect the core coordination sphere. These results establish that ATCUN-type Cu^2+^ binding is not only resistant to chirality inversion but also fully preserved upon sequence reversal, highlighting an exceptional robustness of this metal-binding motif.

### Comparison of Metal Binding Affinities

Competition diagrams were prepared based on potentiometric data to compare the relative metal-binding affinities of the ligands. The diagrams illustrate a hypothetical situation in which equimolar amounts of reagents are mixed, enabling a comparison of the affinity of peptides for a given metal ion.

For Zn^2+^ complexes, all three ligands exhibit nearly identical stability across the investigated pH range (Figure 5). This indicates that neither chirality inversion (L2) nor combined sequence reversal and stereochemical modification (L3) significantly affect Zn^2+^ binding affinity, consistent with the preservation of a histidine-based coordination mode.

**Figure 5:**
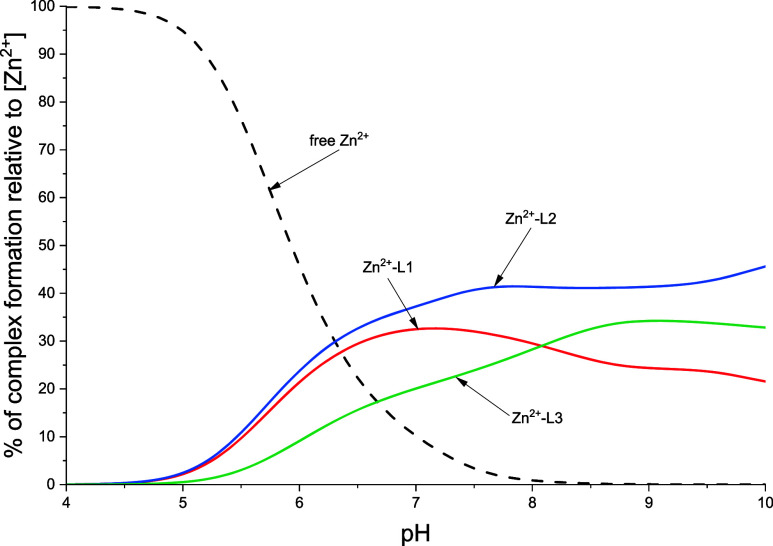
Competition plot between L1 (VFHLLGKIIHHVGNFVYGFSHVF), L2 (vfhllgkiihhvgnfvygfshvf), L3 (fvhsfgyvfngvhiikgllhfv) and Zn^2+^ describing complex formation at different pH values in a hypothetical situation, in which equimolar amounts of all reagents are mixed.

The slightly lower stability of Zn^2+^–L1 relative to other peptides from the clavanin family (A, B, D and E) has previously been attributed to the presence of a peptide backbone O=C–N–H fragment located beneath the metal ion, which weakens the binding site.^5^ Similar stabilities of investigated complexes suggest that the O=C–N–H fragment is also present in coordination sphere of Zn^2+^ in peptidomimetic systems.

In contrast to Zn^2+^, the competition diagram for Cu^2+^ complexes indicates a lower metal-binding affinity of the *retro-inverso* analogue (L3), particularly under alkaline conditions (Figure 6). This indicates that, although the ATCUN coordination mode is preserved, its thermodynamic stability is sensitive to subtle variations in the local sequence environment. A key difference lies in the arrangement of residues surrounding the coordinating histidine. In L1 and L2, the ATCUN motif is embedded in an Xaa–Phe–His sequence, whereas in L3 it adopts a Phe–Xaa–His arrangement. In the former case, the aromatic ring of phenylalanine can engage in stabilizing π–π interactions with the histidine imidazole, facilitated by favorable spatial proximity and orientation. Such interactions are expected to be weaker or less accessible in the *retro-inverso* analogue due to altered backbone geometry and increased distance between the aromatic systems.^39^ We propose that such interactions in the Xaa–Phe–His sequence (present in L1 and L2) are stronger than in the Phe–Xaa–His arrangement (L3), due to the shorter distance between the aromatic systems, allowing more effective interaction and consequently greater stabilization of the Cu^2+^ complex. These observations indicate that, while the ATCUN motif retains its structural integrity under extreme stereochemical perturbations, its stability can be modulated by second-sphere interactions arising from the local sequence context.

**Figure 6:**
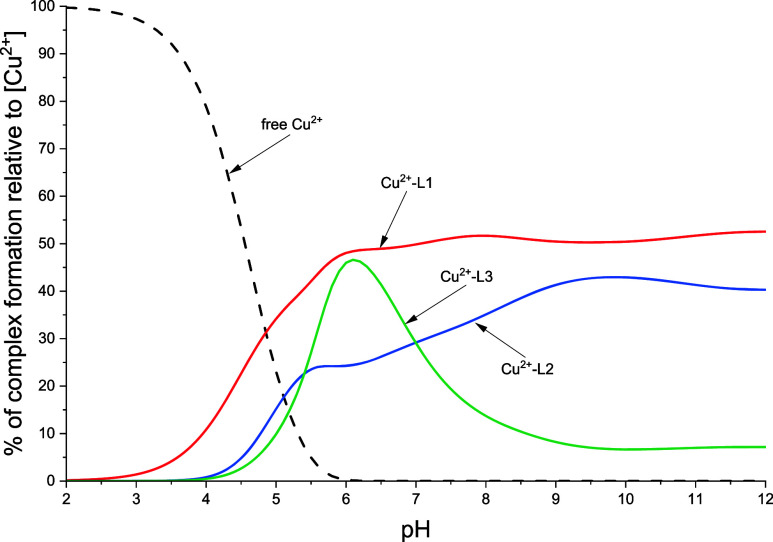
Competition plot between L1 (VFHLLGKIIHHVGNFVYGFSHVF), L2 (vfhllgkiihhvgnfvygfshvf), L3 (fvhsfgyvfngvhhiikgllhfv) and Cu^2+^ describing complex formation at different pH values in a hypothetical situation, in which equimolar amounts of all reagents are mixed.

Taken together, these results reveal two fundamentally different design principles governing metal binding in clavanin C derivatives: Zn^2+^ coordination is dictated by the spatial arrangement of histidine residues and is largely insensitive to sequence identity, whereas Cu^2+^ binding is encoded at the sequence level through the ATCUN motif, which remains structurally robust yet thermodynamically tunable.

### Proteolytic Stability of Native, Retro-Inverso and d-Amino Acid Variants of Clavanin C upon Trypsin Digestion

To evaluate whether stereochemical modification improves resistance to enzymatic degradation, we performed a trypsin digestion experiment on native clavanin C (L1) and its analogues (L2 and L3). Trypsin was selected as a model protease due to its well-defined specificity toward lysine and arginine residues, which are abundant in clavanin C, providing a stringent test of proteolytic stability.

The native peptide undergoes rapid proteolytic degradation, as evidenced by a sharp decrease in the intensity of the parent peak accompanied by the appearance of cleavage products (with retention times: 4.15 and 3.82). Within the first minute, the L1 signal decreased to approximately 56%, and after 30 min the peptide was no longer detectable (Figures S8–S12, S17).

In contrast, both the d-amino acid (L2) and *retro-inverso* (L3) analogues showed complete resistance to trypsin digestion. No additional peaks corresponding to degradation products were observed, and the parent peptide signals remained unchanged even after 30 min of incubation (Figures S13–S16).

These results demonstrate a dramatic enhancement of proteolytic stability upon stereochemical modification. Notably, the *retro-inverso* analogue combines complete resistance to enzymatic degradation with preserved metal-binding properties, highlighting its potential as a robust metallopeptidomimetic.

### Antimicrobial Activity and Cytotoxicity

The antimicrobial activity of clavanin C peptidomimetics and their metal complexes was evaluated against representative reference strains, including two Gram-positive bacteria (*E. faecalis* and methicillin-resistant *S. aureus*), two Gram-negative bacteria (*E. coli* and *P. aeruginosa*), and one yeast species (*C. albicans*). Obtained MIC values were compared with already published results of native clavanin C and their Zn^2+^ and Cu^2+^ complexes.^5^ The investigated mimetics and their metal complexes demonstrated very promising antimicrobial potential, with MIC values comparable to or even lower than those of some currently used antimicrobial agents (Table S1). The results are summarized in Table 2. Dose–response plots (growth vs concentration) for strains showing measurable antimicrobial activity (MRSA and *E. faecalis*) are provided in the Supporting Information (TableS S18 and S19). The highest antibacterial activity of the studied peptidomimetics was observed against the MRSA strain (*S. aureus* ATCC 43300) – for this pathogen, the *retro-inverso* analogue (L3) and its Cu^2+^ and Zn^2+^ complexes were the most active systems, all displaying MIC values of 8 μg/mL. These values are markedly improved relative to the native peptide system (16-fold lower for the free ligand and its Cu^2+^ complex, and 2-fold lower for the Zn^2+^ complex, compared to the native peptide system (L1 and L1–Cu^2+^)). The d-amino acid mimetic (L2) and its metal complexes (MIC = 32 μg/mL) showed 4-fold higher activity than native clavanin C (L1) and the L1–Cu^2+^ complex, but were 2-fold less active than the corresponding Zn^2+^ complexes. The findings indicate that d-amino acid based and *retro-inverso* redesign not only preserves, but can also enhance antimicrobial performance against a clinically relevant resistant strain.

**Table 2:** Results of Antimicrobial Activity Tests of Clavanin C, its d-Amino Acid and Retro-inverso Mimetics and Their Complexes with Zn^2+^ and Cu^2+^ Against Reference Strains, Expressed as MIC/MBC Values [μg/mL]; n/d – No Activity Detected^a^

	*E. coli* (−) ATCC 25922	*E. faecalis* (+) ATCC 29212	*S. aureus* (+) ATCC 43300	*C. albicans* ATCC 10231
	MIC [μg/mL]	MIC [μg/mL]	MIC [μg/mL]	MIC [μg/mL]
Clavanin C (L1)^5^	n/d	128	128	64
Cu^2+^-L1	128	256	128	64
Zn^2+^-L1	**16**	**64**	**16**	16
D-clavanin C (L2)	n/d	n/d	**32**	n/d
Cu^2+^-L2	n/d	**64**	**32**	n/d
Zn^2+^-L2	n/d	n/d	**32**	n/d
RI-clavanin C (L3)	n/d	**64**	**8**	n/d
Cu^2+^-L3	n/d	n/d	**8**	n/d
Zn^2+^-L3	n/d	**32**	**8**	n/d

a All experiments were performed in accordance with ISO 20776-1:2019 ^25^ and ISO 16256:2021.^26^ No MIC value was determined for Pseudomonas aeruginosa ATCC 27853. No MBC/MFC activity was observed after performing modified Richard’s method.^27,28^ Bolded values represent concentrations lower than or equal to EUCAST breakpoints (Table S1) for selected antimicrobial agents characteristic for given bacterial families.

Notably, for MRSA, metal coordination did not substantially alter the activity of the peptidomimetics, suggesting that the intrinsic properties of the modified peptide scaffold dominate the biological response in this case. A different trend was observed for *E. faecalis*, where metal coordination had a more pronounced effect on activity. In the case of L2, Cu^2+^ complexation was required for the emergence of detectable antimicrobial activity, whereas the free peptide remained inactive. For the *retro-inverso* analogue, Zn^2+^ coordination enhanced activity 2-fold, lowering the MIC from 64 to 32 μg/mL. These results indicate that, although the peptidomimetic scaffold itself is important, metal coordination can further modulate antimicrobial potency in a strain-dependent manner. On the other hand, the applied structural modifications led to a loss of antifungal activity against *C. albicans*. A similar trend was observed for the *E. coli* strain, where no antimicrobial activity was detected for the modified analogues and their complexes. This observation may indicate that the structural modifications alter peptide–membrane interactions or target selectivity, favoring activity toward Gram-positive bacteria while reducing effectiveness against fungal and Gram-negative cells.

It should be emphasized that the antimicrobial activity cannot be attributed to the metal ions alone. The MIC values determined for Zn^2+^ and Cu^2+^ salts are provided in Table S2 (adapted from ref­[^38^]) for comparison purposes.

Remarkably, the *retro-inverso* analogue not only preserves metal-binding properties and exhibits complete resistance to proteolytic degradation, but also surpasses the native peptide in antimicrobial activity, particularly against MRSA, establishing it as a superior scaffold for the design of stable and effective metallopeptidomimetics.

The cytotoxicity of all active compounds and their metal complexes was evaluated using the neutral red uptake assay in L929 cells. After 24, 48, and 72 h of incubation, cell viability remained in the range of 85–100%, indicating that the investigated systems are not detectably cytotoxic within the tested concentration range.

## Discussion

The obtained results suggest that antimicrobial activity of clavanin C and its d-amino acid and *retro-inverso* analogues is closely linked to bacterial membrane composition and organization. The higher activity observed against Gram-positive bacteria compared to Gram-negative strains suggests the cytoplasmic membrane as the primary target of these peptides. In Gram-negative bacteria, the outer membrane enriched in lipopolysaccharides limits peptide access to the cytoplasmic membrane, while the lack of anionic phospholipids in the outer membrane such as phosphatidylglycerol and cardiolipin further reduces peptide-lipid interactions. Although increased lysyl-phosphatidylglycerol (Lys-PG) content is generally associated with reduced susceptibility to cationic antimicrobial peptides,^40^ MRSA showed higher sensitivity to clavanin C and its analogues than *E. faecalis*. Lys-PG decreases membrane surface charge and typically electrostatically repels classical antimicrobial peptides; however, clavanin C appears less dependent on electrostatic attraction than classical cationic antimicrobial peptides. The heterogeneous distribution of Lys-PG and MprF-mediated disruption of membrane asymmetry generate local regions of increased membrane tension, which may facilitate peptide-induced destabilization, particularly for peptides that disrupt lipid packing rather than forming defined pores.^41^ In contrast, *E. faecalis* exhibits pronounced lipidomic plasticity and a higher glycolipid content, enabling dynamic membrane remodeling in response to stress.^42,43^ Such adaptability may reduce membrane susceptibility to peptide-induced damage, contributing to the lower activity observed for all clavanin C variants compared to MRSA, despite the presence of PG- and CL-rich membrane regions. The limited impact of Zn^2+^ coordination on antimicrobial activity in the modified peptides suggests that their biological properties depend primarily on precise peptide conformation and, critically, on the biological stability of antimicrobial peptides. One of the key virulence factors of MRSA is the production of proteolytic enzymes, including staphopains and aureolysin, which are capable of degrading and inactivating native AMPs such as cathelicidin LL-37.^44^ In this context, the enhanced activity of the d-amino acid and especially the *retro-inverso* analogue can be directly linked to their complete resistance to proteolytic degradation.

The observed differences between the l-, d-amino acid, and *retro-inverso* forms of clavanin C likely reflect distinct modes of membrane interaction – mimetics of clavanin C may not rely exclusively on pore formation, but rather involve a “carpet-like” mechanism based on the accumulation of antimicrobial peptides on the membrane surface in a parallel orientation, leading to membrane destabilization and disintegration once a critical peptide concentration is reached and formation of micelle-like structures.^45–47^ While substitution with d-amino acids confers resistance to proteolytic degradation, it preserves peptide bond orientation, potentially increasing the effective availability of the peptide at the membrane. In contrast, the *retro-inverso* analogue, characterized by an inverted backbone, may interact with lipid bilayers in a more flexible manner due to inversion of the peptide backbone, favoring large-scale membrane disorganization rather than discrete pore formation. Together, these observations demonstrate that stereochemical redesign of clavanin C provides an effective strategy for the development of proteolytically stable metallopeptidomimetics with enhanced biological performance.

## Conclusion

In this study, we developed enzymatically stable analogues of clavanin C and its metal complexes that combines enhanced antimicrobial potency with low cytotoxicity. By applying all-d amino acid substitution and a *retro-inverso* design, we obtained peptidomimetics that are fully resistant to proteolytic degradation while retaining high biological activity.

Crucially, despite complete stereochemical inversion and sequence reversal, Cu^2+^ coordination is preserved across all systems due to the specific primary structure of clavanin C. The presence of histidine residues in the third position from both the N- and C-terminus preserves the ATCUN motif, enabling the formation of square-planar Cu^2+^ complexes irrespective of peptide directionality. In contrast, Zn^2+^ binding remains largely unaffected by stereochemical modifications, reflecting a coordination mode governed primarily by the spatial clustering of histidine side chains rather than backbone orientation.

Importantly, all peptidomimetics and their metal complexes retain an α-helical secondary structure under membrane-mimicking conditions, comparable to that of the native peptide. Preservation of this helical fold ensures a similar spatial arrangement of functional side chains, providing a structural basis for the maintained metal-binding properties and biological activity.

These chemically and structurally conserved features translate directly into biological performance. In particular, the *retro-inverso* analogue and its Cu^2+^ and Zn^2+^ complexes exhibit remarkably strong and selective activity against MRSA, surpassing the native peptide while maintaining low cytotoxicity. Overall, this study demonstrates that deliberate stereochemical redesign can be used to obtain enzymatically stable clavanin C-based peptidomimetics without compromising their secondary structure, metal-binding competence, or biological performance. By exploiting the specific placement of histidine residues within the sequence, Cu^2+^ coordination via an ATCUN-type motif is retained even upon sequence reversal and inversion of chirality, while Zn^2+^ binding remains governed by histidine clustering within the α-helical fold. These findings provide a rational framework for designing stable antimicrobial metallopeptides in which proteolytic resistance, metal coordination, and biological activity can be optimized simultaneously.

## Supplementary Material



## Notes

ESI-MS spectra and MS spectra of Zn­(II)-clavanin C (L1), Zn­(II)-Daa-clavanin C (L2), and Zn­(II)-retro-inverso clavanin C (L3) samples; MS spectra of Zn­(II)-clavanin C (L1), Zn­(II)-Daa-clavanin C (L2), and Zn­(II)-retro-inverso clavanin C (L3) samples; distribution diagrams for the formation of Zn­(II) complex with clavanin C, Zn­(II) complex with Daa-clavanin C, and Zn­(II) complex with RI-clavanin C; distribution diagrams for the formation of Cu­(II) complex with clavanin C, Cu­(II) complex with Daa-clavanin C, and Cu­(II) complex with RI-clavanin C; UV–vis spectra of Cu­(II) complexes with clavanin C, Daa-clavanin C and RI-clavanin C in pH range 3–11; CD spectra of Cu­(II) complexes with clavanin C, Daa-clavanin C and RI-clavanini C in pH range 3–11; secondary structure of clavanin C, Daa-clavanin C, RI-clavanin C and their copper­(II) and zinc­(II) complexes; and examples of MIC breakpoints values from EUCAST Version 14.0 for bacteria (PDF)
