# Supplementary material for: Stereochemical Control of Cu(II) and Zn(II) Binding in Clavanin C Peptidomimetics
Source: Inorg Chem. 2026 Jun 25;65(27):15845–54. doi: 10.1021/acs.inorgchem.6c02172 (PMC13370850; doi:10.1021/acs.inorgchem.6c02172)
Supplement: Supplementary file 1 [file ic6c02172_si_001.pdf]

Supplementary information

Stereochemical control of Cu(II) and Zn(II) binding in clavanin C peptidomimetics

Jakub Gawłowski,[1] Mariusz Dziadas,[1] Aleksandra Mikołajczyk-Tarnawa,[2] Arian Kola,[3] Daniela Valensin,[3,4] Agnieszka Matera-Witkiewicz,[2] Magdalena Rowińska-Żyrek*[1]

[1] *Faculty of Chemistry, University of Wrocław, F. Joliot-Curie 14, 50-383 Wrocław, Poland; email: magdalena.rowinska-zyrek@uwr.edu.pl*

[2] *Screening Laboratory of Biological Activity Tests and Collection of Biological Material, Faculty of Pharmacy, Wroclaw Medical University, Borowska 211A, 50-556 Wroclaw, Poland;*

[3] *Department of Biotechnology, Chemistry and Pharmacy, University of Siena, Via A. Moro 2, 53100 Siena, Italy;*

[4] *CIRMMP, Via Luigi Sacconi 6, 50019 Sesto Fiorentino, Italy.*

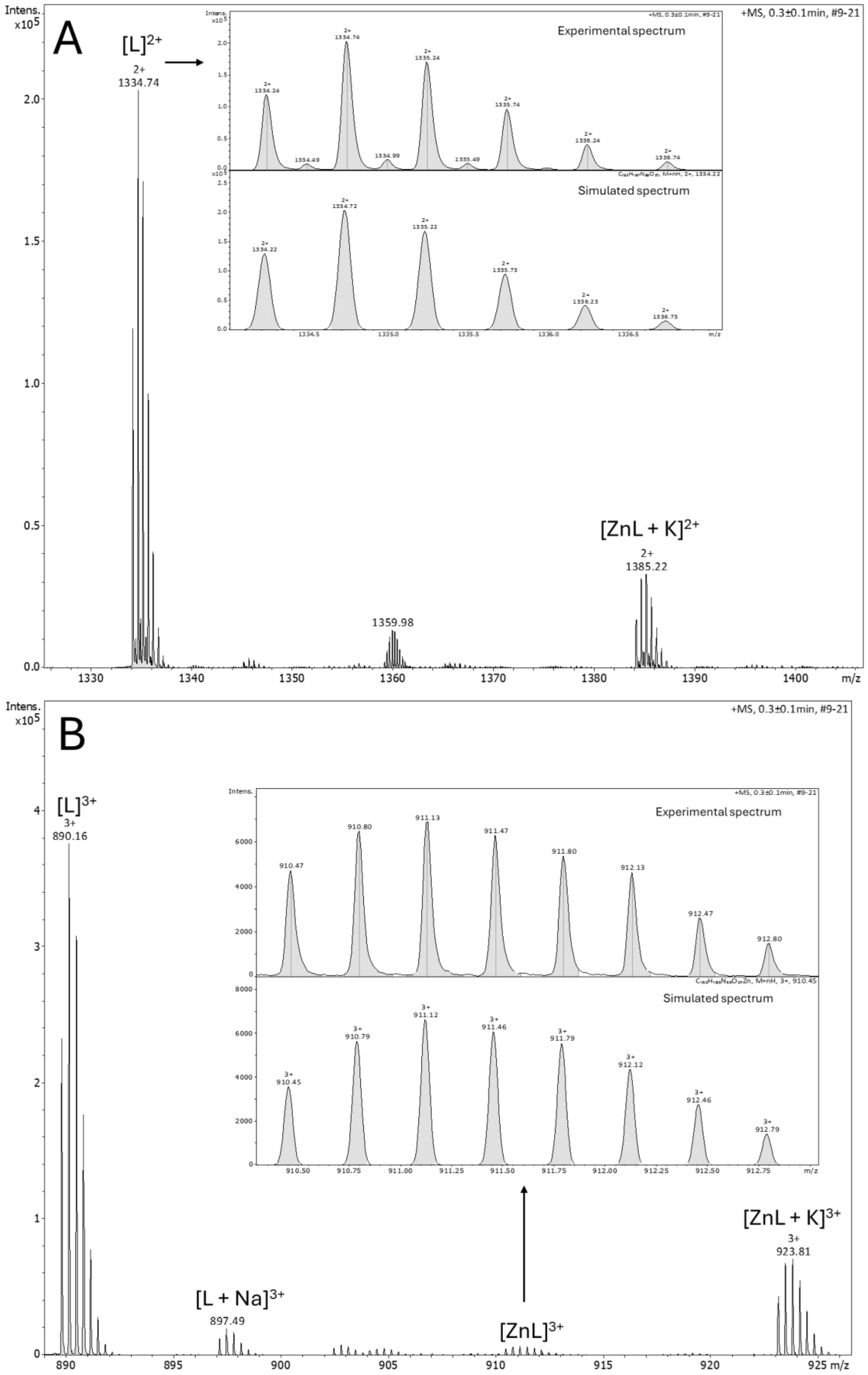
A
Intens.
x10^5
[L]2+
2+
1334.74
+MS, 0.3±0.1min, #9-21
Experimental spectrum
Simulated spectrum
[ZnL + K]2+
2+
1385.22
1359.98
m/z
B
Intens.
x10^5
+MS, 0.3±0.1min, #9-21
[L]3+
3+
890.16
Experimental spectrum
Simulated spectrum
[L + Na]3+
897.49
[ZnL]3+
[ZnL + K]3+
3+
923.81
m/z

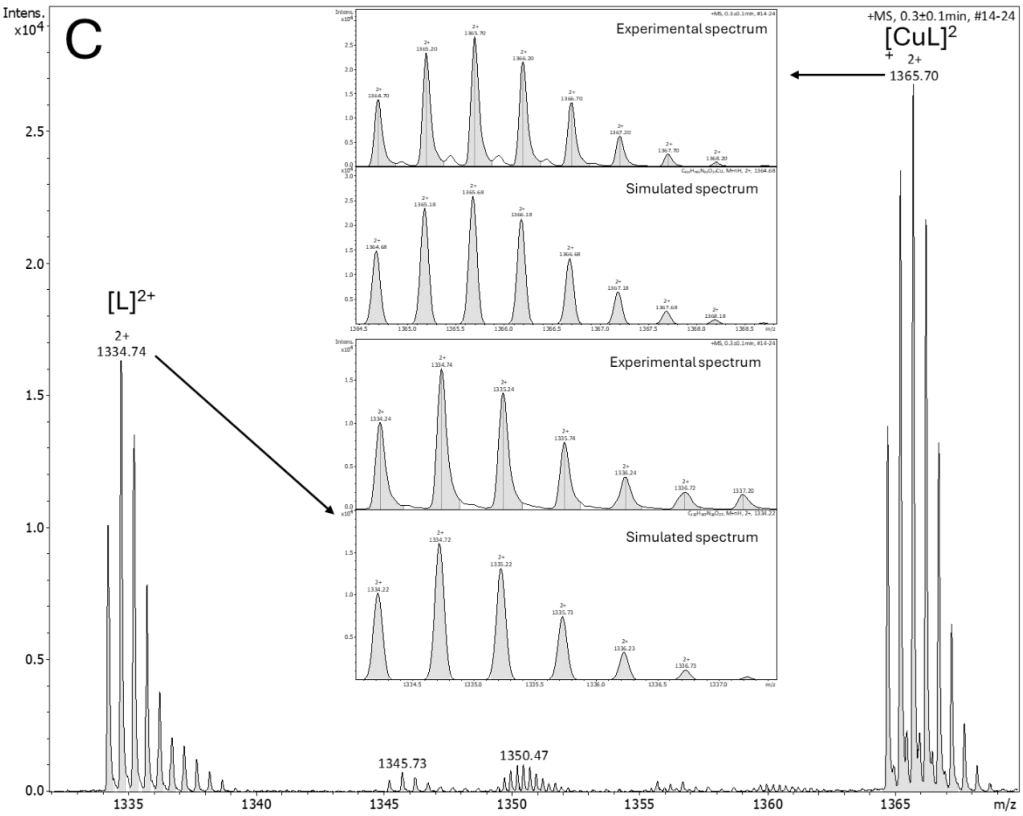
C
Intens. x10^4
+MS, 0.3±0.1min, #14-24
[CuL]2+
2+
1365.70
[L]2+
2+
1334.74
1345.73
1350.47
Experimental spectrum
Simulated spectrum
m/z

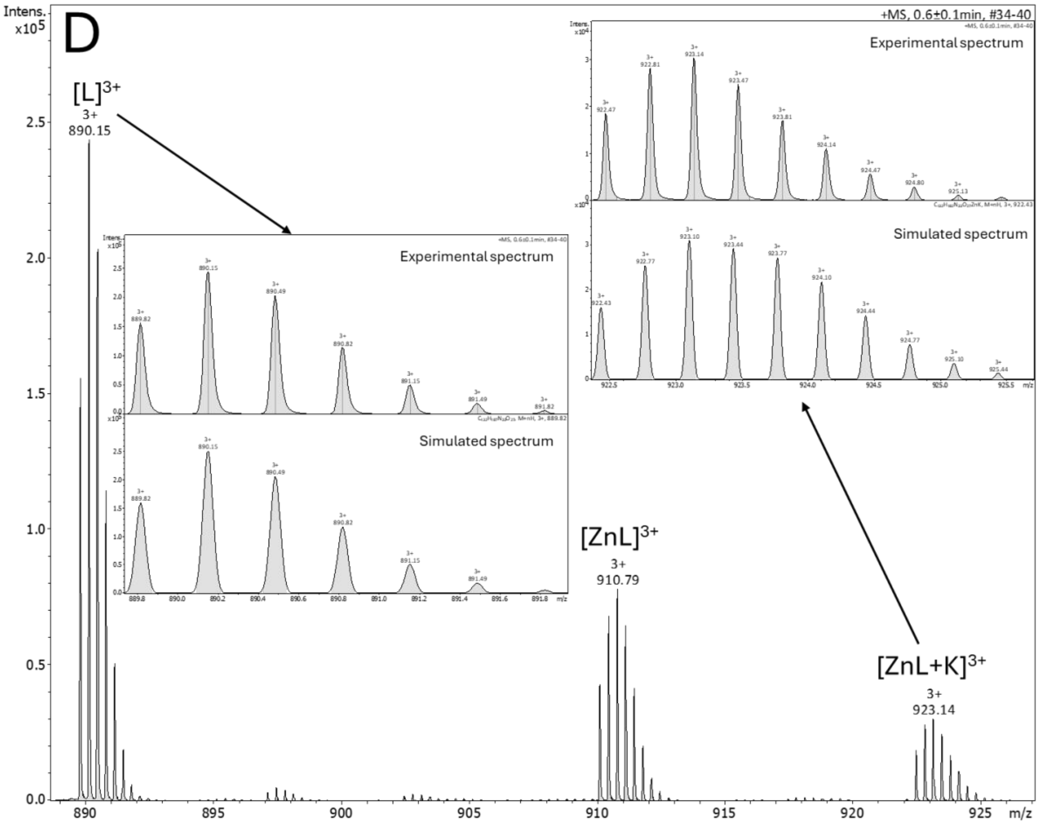
D
Intens. x10^5
+MS, 0.6±0.1min, #34-40
[L]3+
3+
890.15
[ZnL]3+
3+
910.79
[ZnL+K]3+
3+
923.14
Experimental spectrum
Simulated spectrum
m/z

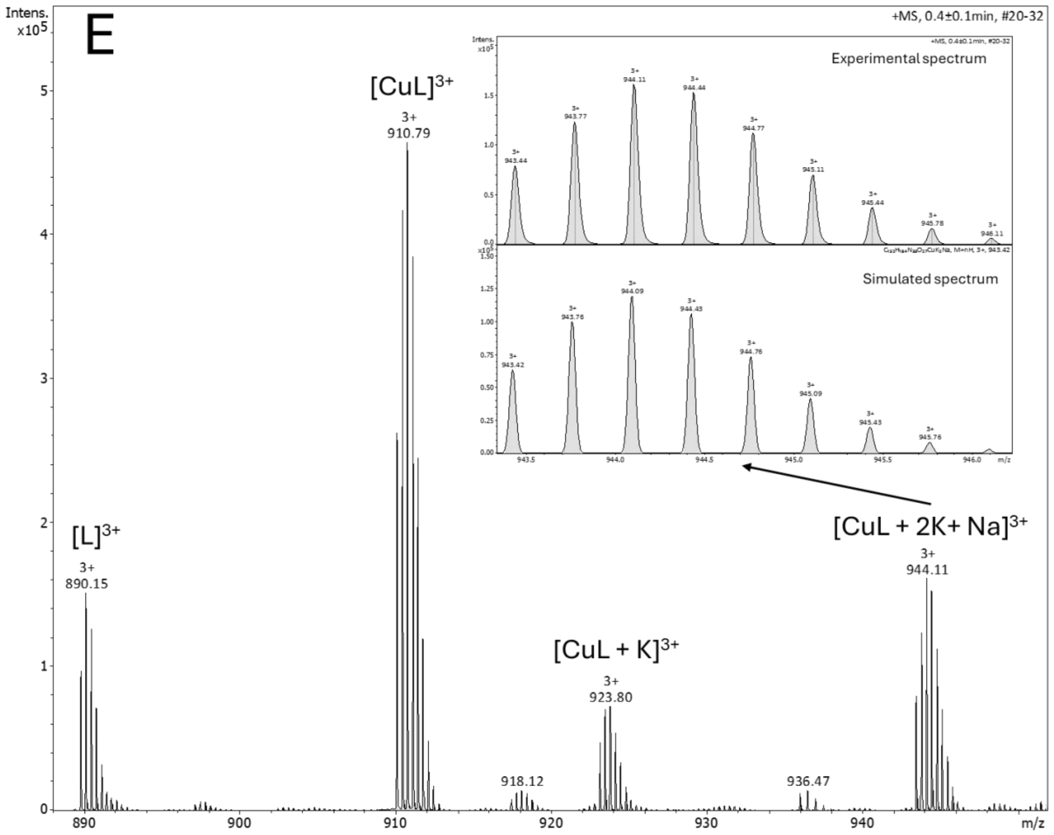
E
Intens.
x10^5
+MS, 0.4±0.1min, #20-32
[CuL]3+
3+
910.79
[L]3+
3+
890.15
[CuL + K]3+
3+
923.80
918.12
936.47
[CuL + 2K+ Na]3+
3+
944.11
Experimental spectrum
Simulated spectrum
m/z

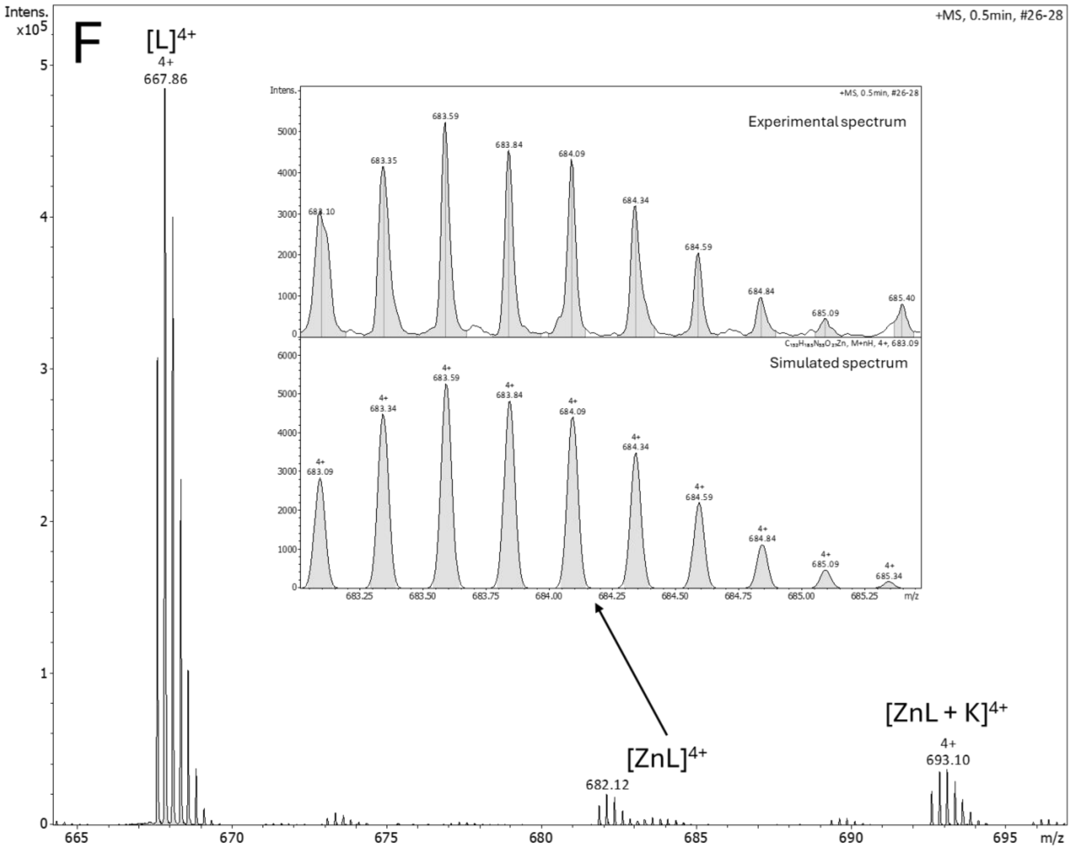
F
Intens.
x10^5
+MS, 0.5min, #26-28
[L]4+
4+
667.86
Experimental spectrum
Simulated spectrum
[ZnL]4+
682.12
[ZnL + K]4+
4+
693.10
m/z

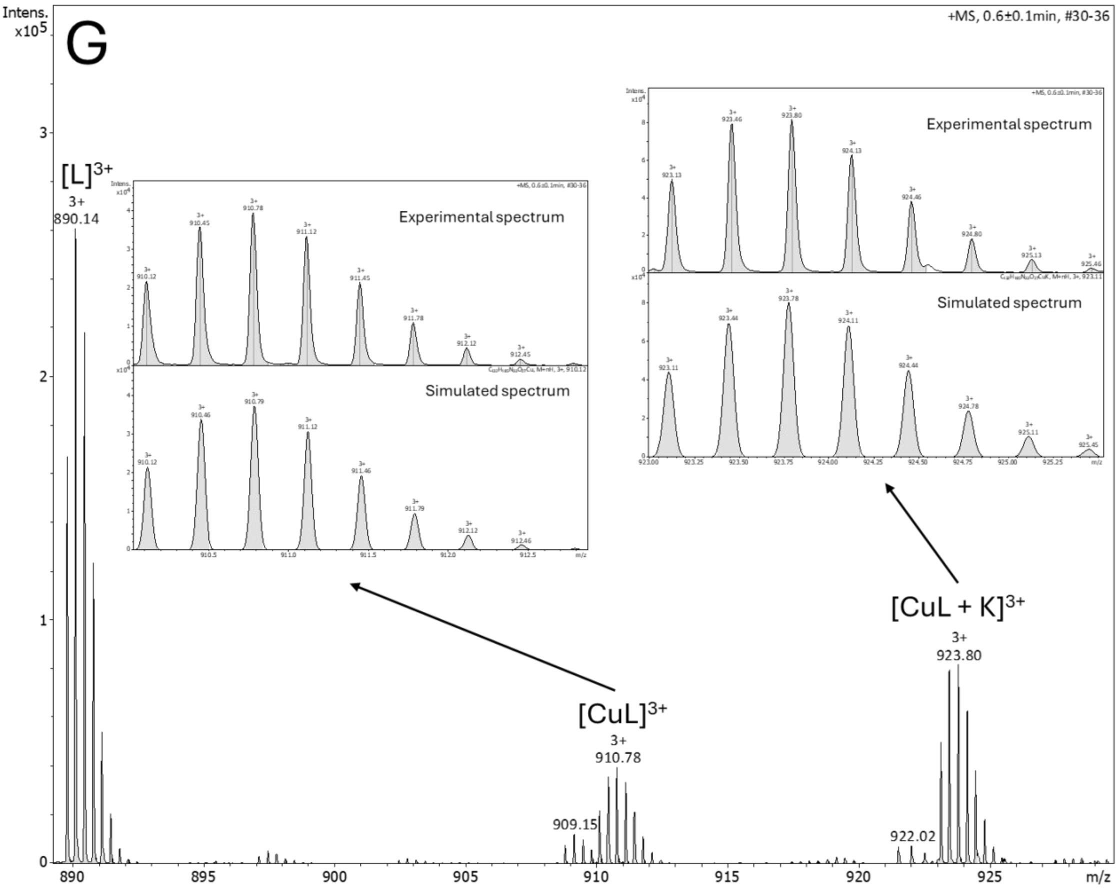

Figure S1. ESI-MS spectra for A) and B) Zn^{2+} complex with L1 (VFHLLGKIIHHVGNFVYGFSHVF); C) Cu^{2+} complex with L1; D) Zn^{2+} complex with L2 (vfhllgkiihhvgnfvygfshvf); E) Cu^{2+} complex with L2; F) Zn^{2+} complex with L3 (fvhsfgyvfngvhhiikgllhfv) and G) Cu^{2+} complex with L3 with enlarged chosen m/z regions. Comparisons of experimental and simulated spectra are presented for selected signals. Molar ratio M:L – 1:1. [L] = 0.0001 M. Samples prepared in MeOH:H_2O (50:50) mixture.

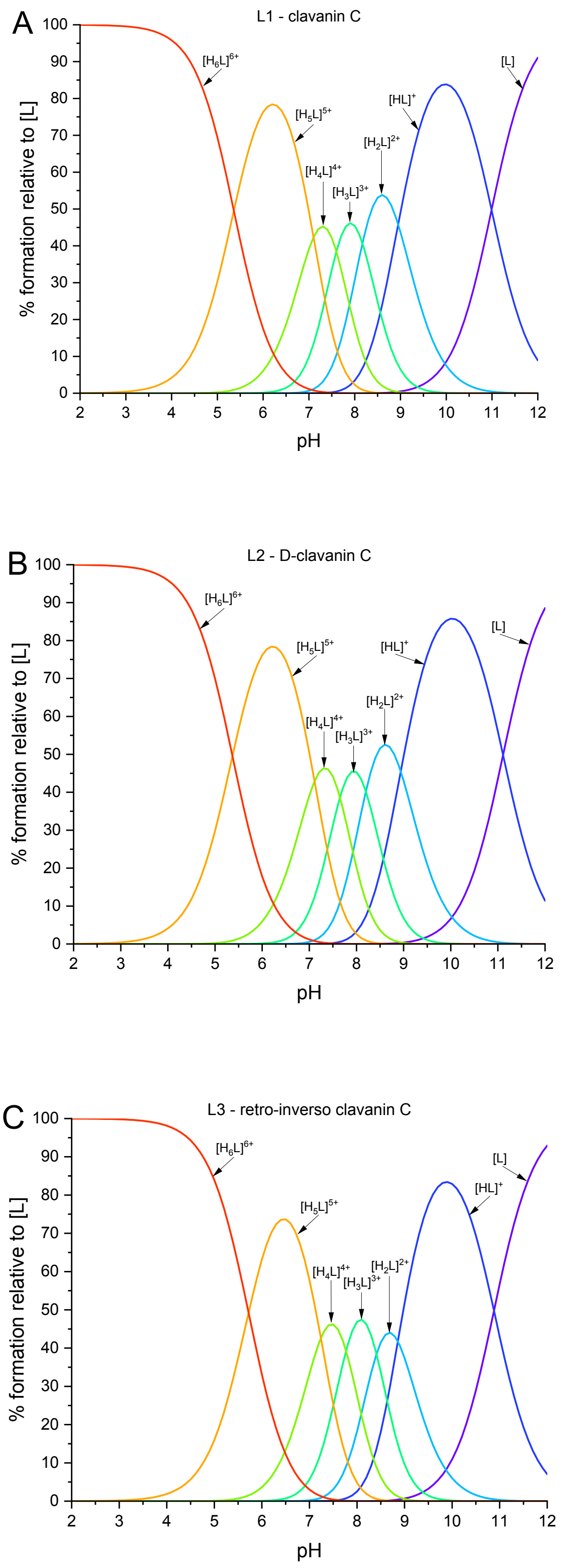

Figure S2. Representative distribution diagram for (A) L1 (VFHLLGKIIHHVGNFVYGFSHVF); (B) L2 (vfhllgkiihhvgnfvygfshvf) and (C) L3 (fvhsfgyvfngvhhiikgllhfv) peptides in solution of 0.004 M HClO4 with I = 40 mM SDS dependent on pH values. [L] = 0.0004 M.

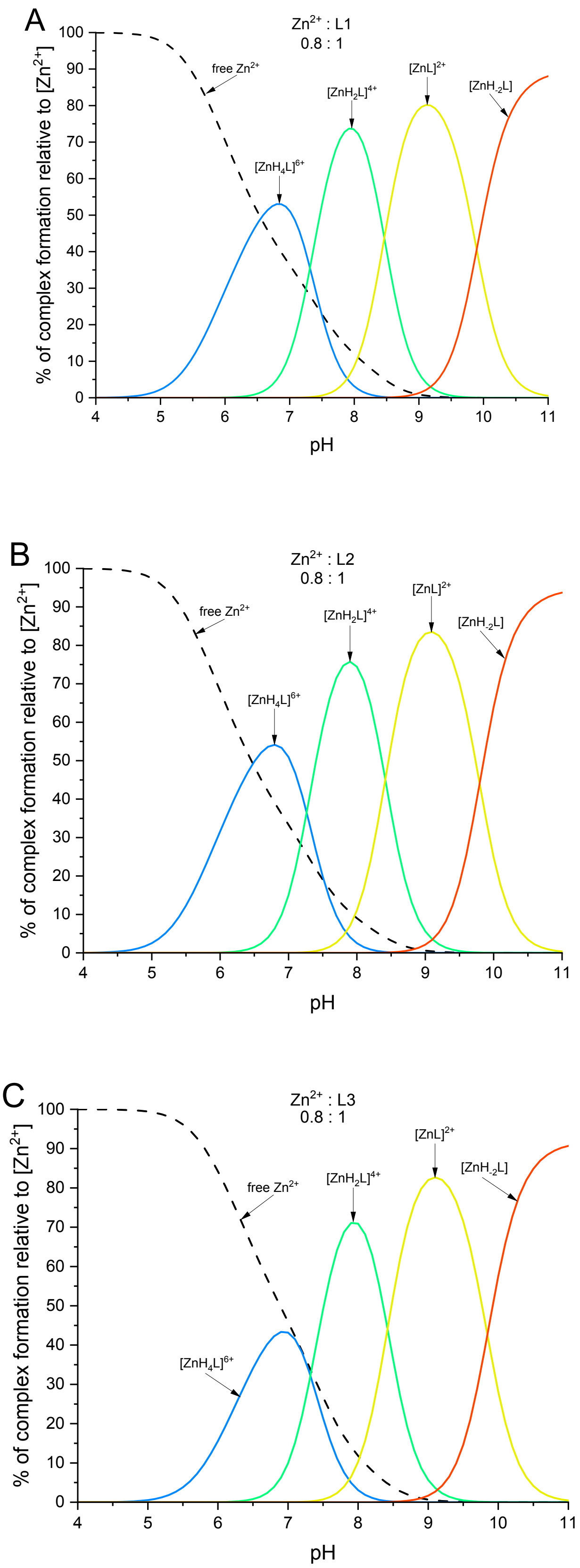

Figure S3. Representative distribution diagram for (A) Zn^{2+}–L1 (VFHLLGKIIHHVGNFVYGFSHVF); (B) Zn^{2+}–L2 (vfhllgkiihhvgnfvygfshvf) and (C) Zn^{2+}–L3 (fvhsfgyvfngvhhiikgllhfv) systems in solution of 0.0004 M $HClO_4$ with I = 40 mM SDS. [L] = 0.4 mM; molar ratio M:L – 0.9:1. T = 25 °C.

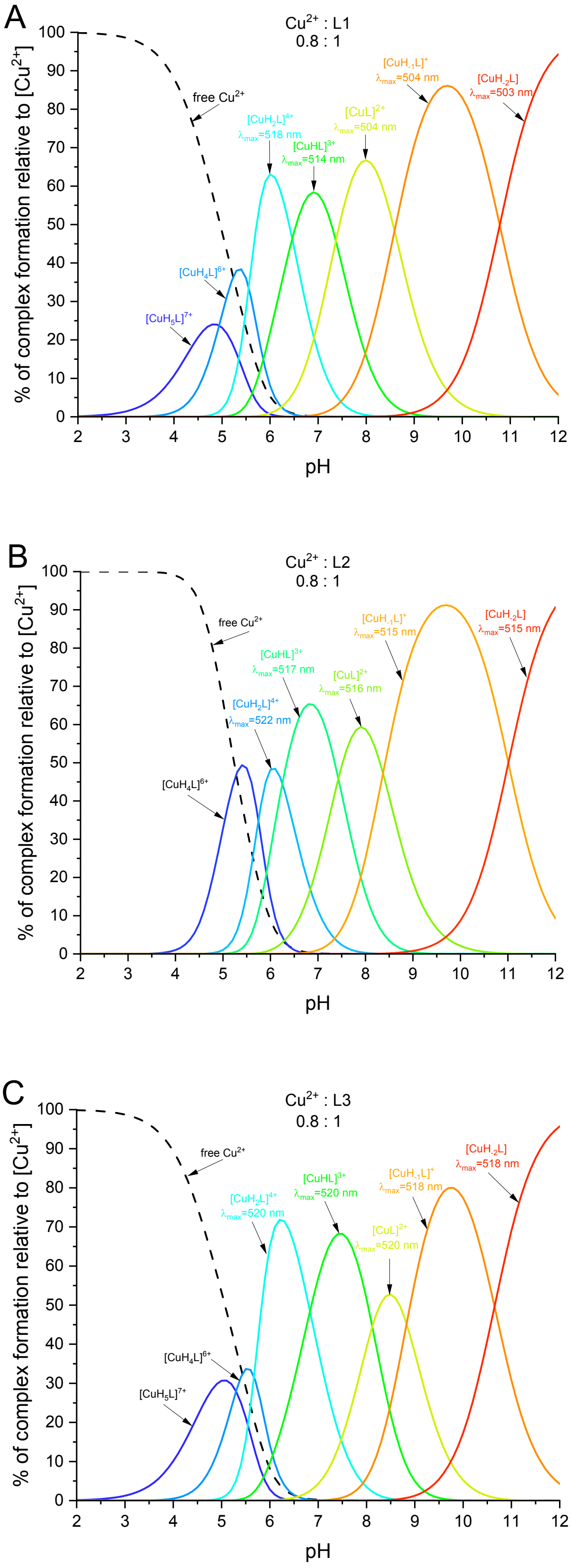

Figure S4. Representative distribution diagram for (A) Cu^{2+}–L1 (VFHLLGKIIHHVGNFVYGFSHVF); (B) Cu^{2+}–L2 (vfhllgkiihhvgnfvygfshvf) and (C) Cu^{2+}–L3 (fvhsfgyvfngvhhiikgllhfv) systems in solution of 0.0004 M $HClO_4$ with I = 40 mM SDS. [L] = 0.4 mM; molar ratio M:L – 0.8:1. T = 25 °C.

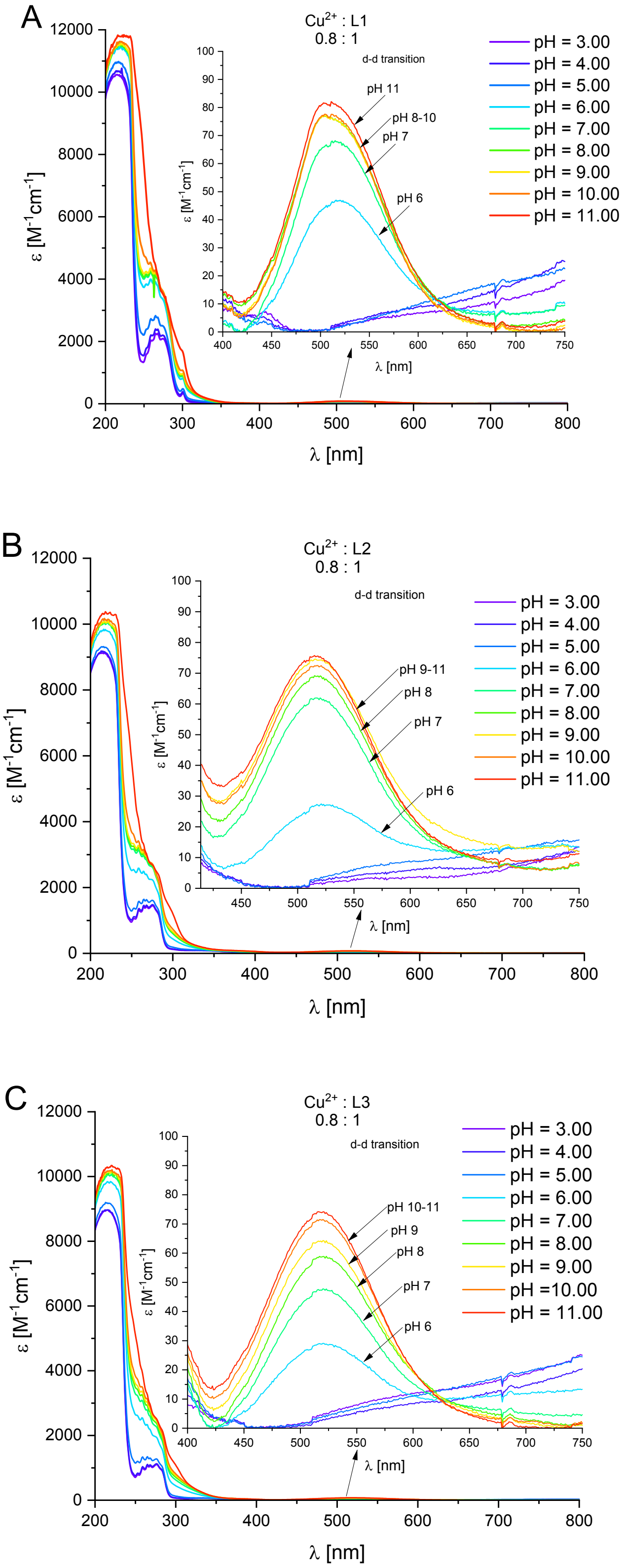

Figure S5. UV-Vis spectra for (A) Cu^{2+}–L1 (VFHLLGKIIHHVGNFVYGFSHVF), (B) Cu^{2+}–L2 (vfhllgkiihhvgnfvygfshvf), and (C) Cu^{2+}–L3 (fvhsfgyvfngvhhiikgllhfv) complexes. The molar ratio of M:L is 0.8:1, with [L] = 0.4 mM. Measurements were conducted in 0.0004 M $HClO_4$ solution with I = 40 mM SDS, at T = 298 K; optical path length (l) = 1 cm.

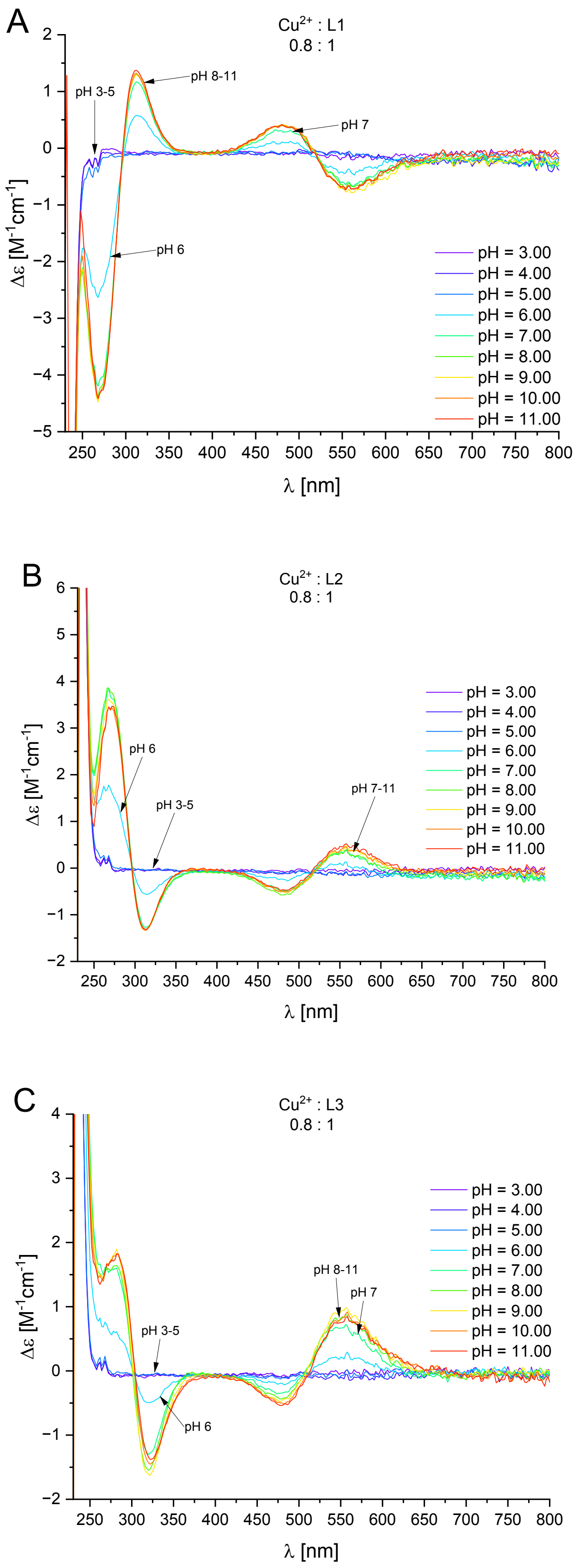

Figure S6. Circular dichroism (CD) spectra for (A) Cu^{2+}–L1 (VFHLLGKIIHHVGNFVYGFSHVF), (B) Cu^{2+}–L2 (vfhllgkiihhvgnfvygfshvf), and (C) Cu^{2+}–L3 (fvhsfgyvfngvhhiikgllhfv) complexes. The molar ratio of M:L is 0.8:1, with [L] = 0.4 mM. Measurements were conducted in 0.0004 M $HClO_4$ solution with I = 40 mM SDS, at T = 298 K; optical path length (l) = 1 cm.

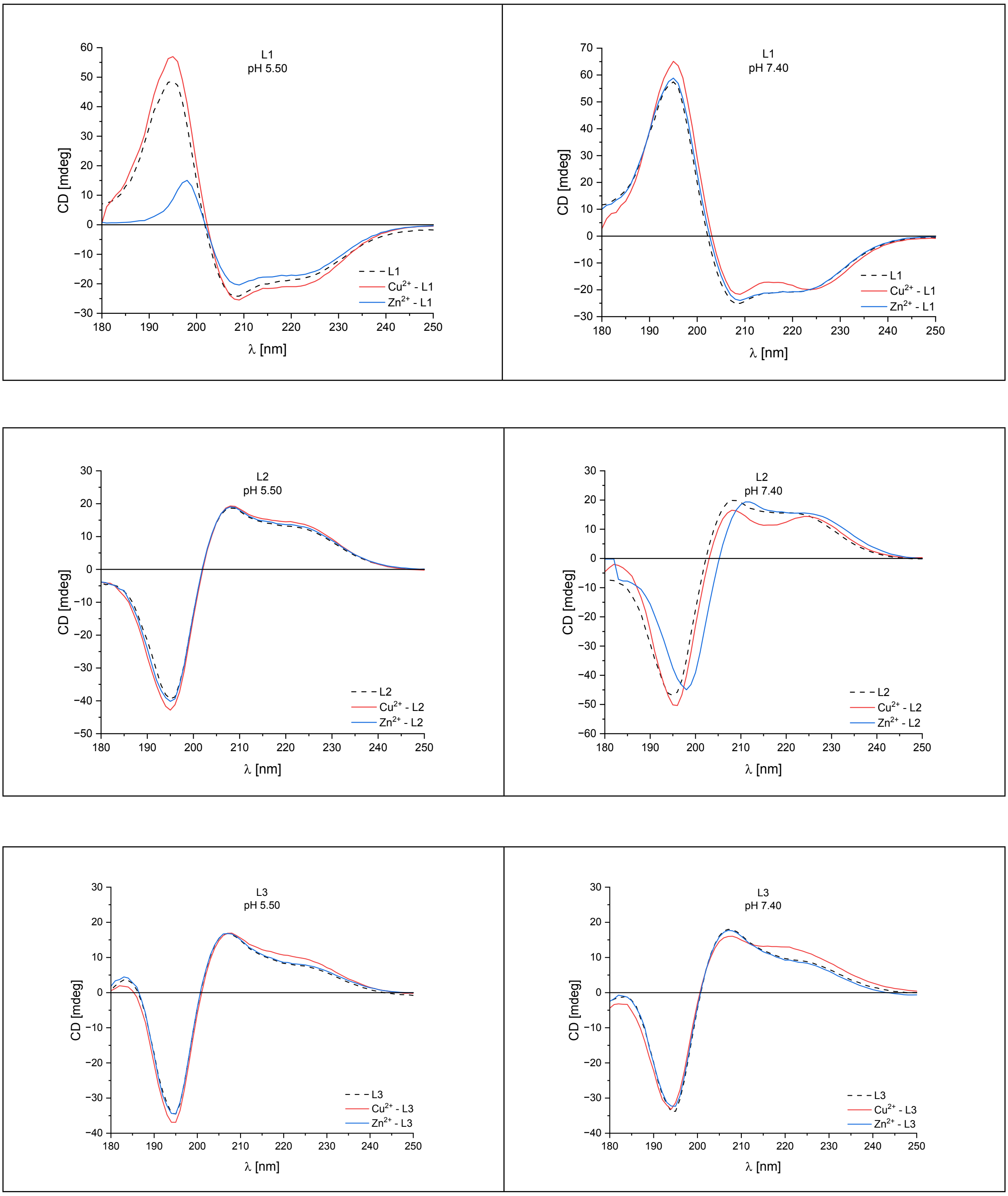

Figure S7. Far-UV CD spectra of clavanin C (VFHLLGKIIHHVGNFVYGFSHVF) and its investigated mimetics (D-amino acid mimetic: vfhllgkiihhvgnfvygfshvf and retro-inverso analog: fvhsfgyvfngvhhiikgllhfv) and their complexes with Cu^{2+} and Zn^{2+}. The molar ratio of M:L is 0.8:1, with [L] = 0.4 mM. Measurements were conducted in 0.0004 M $HClO_4$ solution with I = 40 mM SDS, at T = 298 K; optical path length (l) = 0.2 cm.

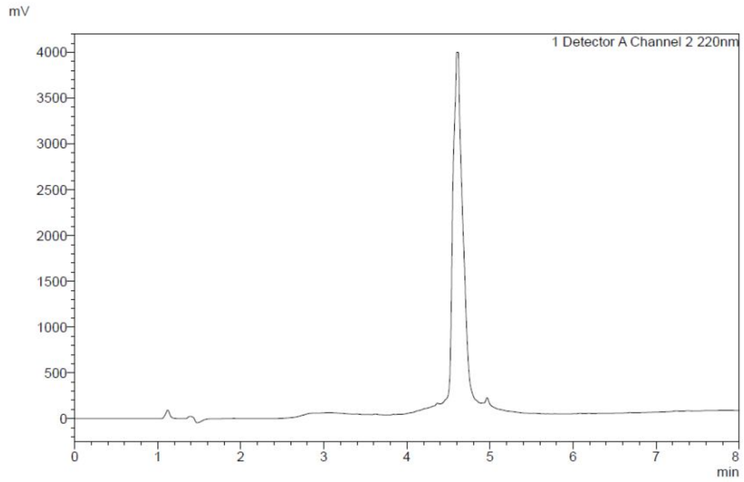

Figure S8. HPLC-UV (220nm) chromatogram of clavanin C (L1) standard (undigested).

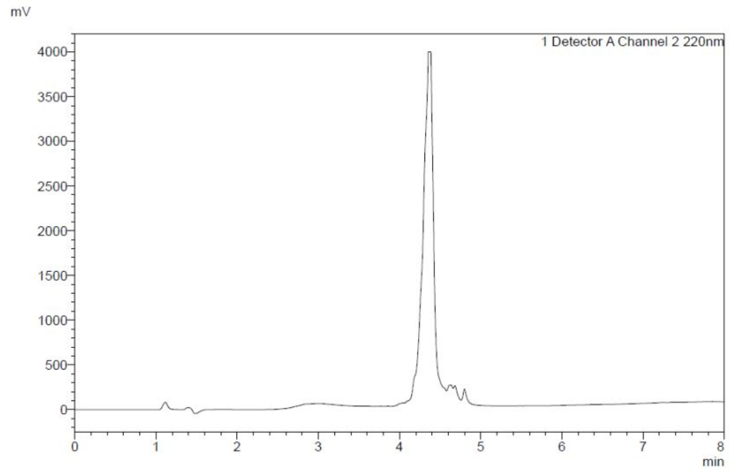

Figure S9. HPLC-UV (220nm) chromatogram of D-clavanin C (L2) standard (undigested).

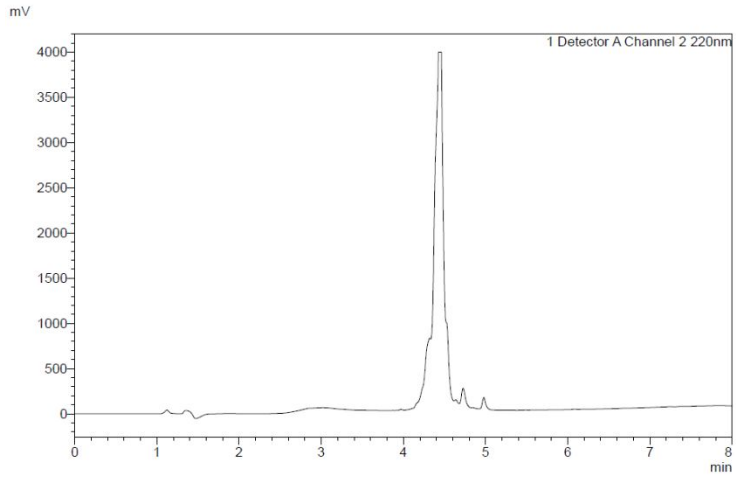

Figure S10. HPLC-UV (220nm) chromatogram of RI-clavanin C (L3) standard (undigested).

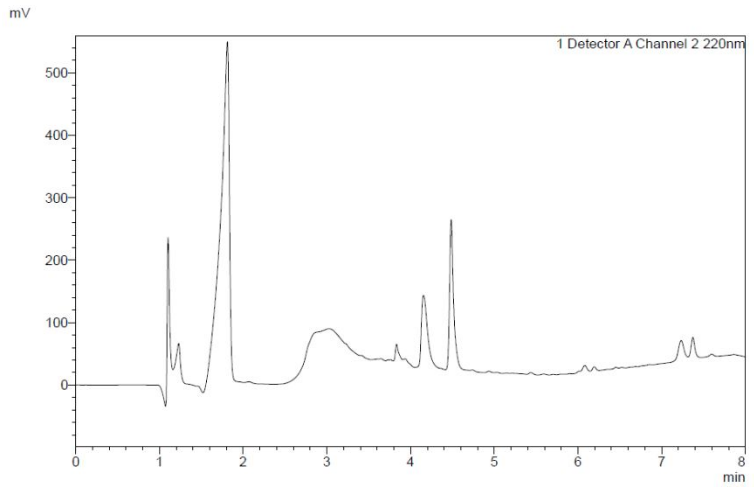

Figure S11. HPLC-UV (220 nm) chromatogram of clavanin C (L1) after 1 minute of incubation. Corresponding peak area: 4543590.

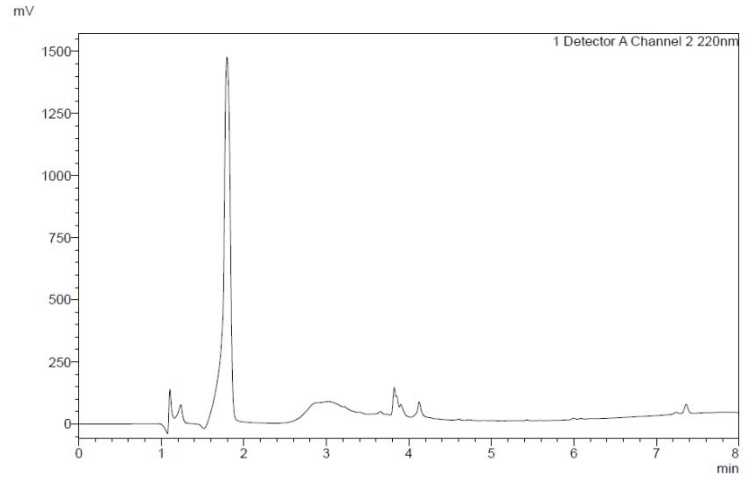

Figure S12. HPLC-UV (220 nm) chromatogram of clavanin C (L1) after 30 minutes of incubation with trypsin. Peak area corresponding to native peptide: 0.

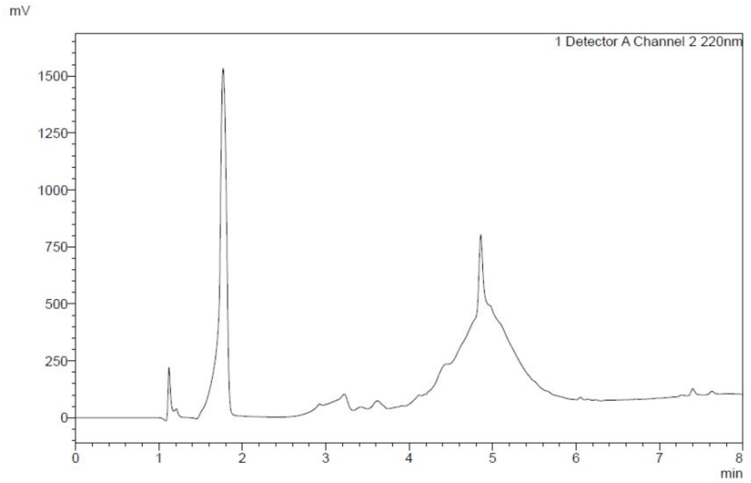

Figure S13. HPLC-UV (220 nm) chromatogram of D-clavanin C (L2) after 1 minute of incubation with trypsin. Corresponding peak area: 1003621.

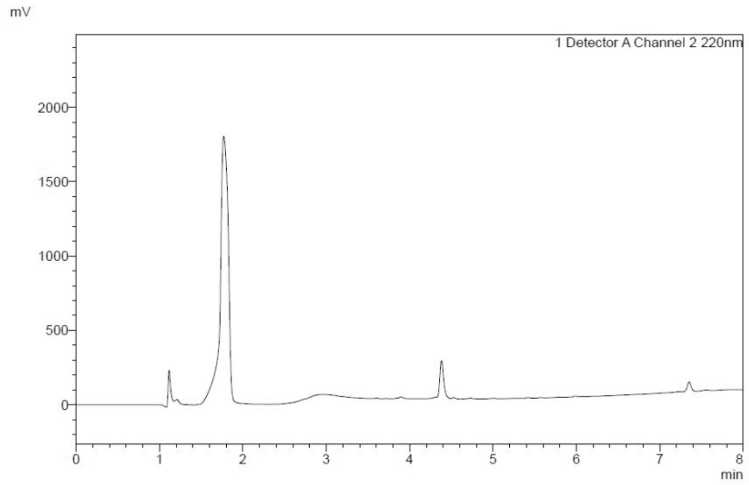

Figure S14. HPLC-UV (220 nm) chromatogram of D-clavanin C (L2) after 30 minutes of incubation with trypsin. Peak area corresponding to native peptide: 1001927.

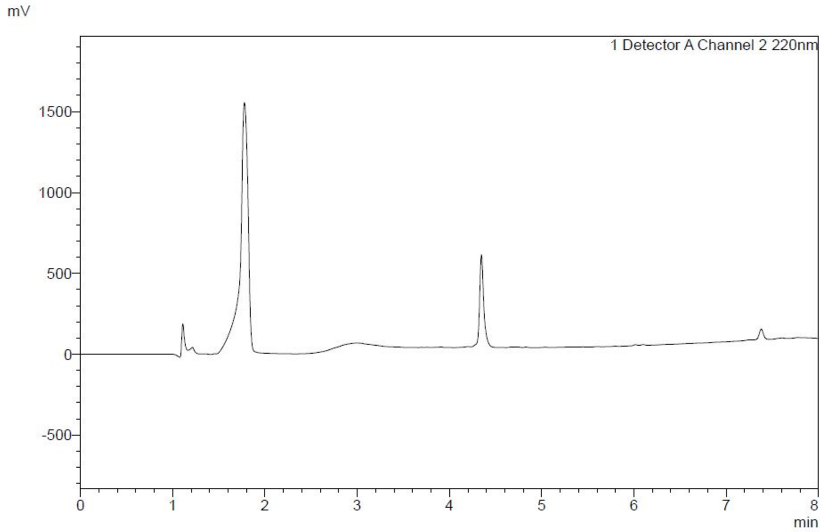

Figure S15. HPLC-UV (220 nm) chromatogram of RI-clavanin C (L3) after 1 minute of incubation with trypsin. Corresponding peak area: 1642735.

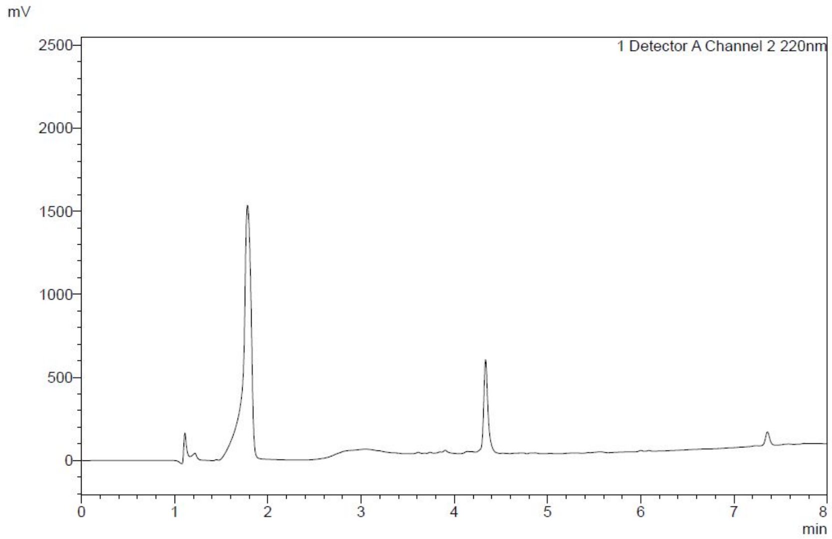

Figure S16. HPLC-UV (220 nm) chromatogram of RI-clavanin C (L3) after 30 minutes of incubation with trypsin. Peak area corresponding to native peptide: 1679279.

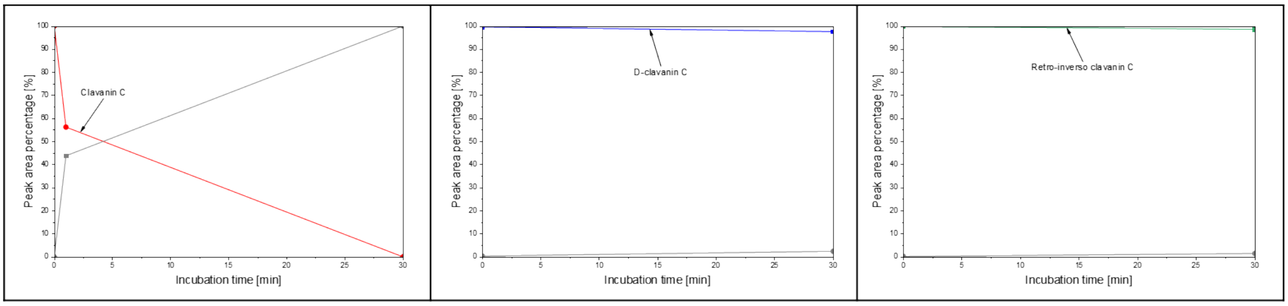

Figure S17. Changes in the percentage contribution of clavanin C peptide (red), D-amino acid clavanin C (blue), retro-iverso clavanin C (green) and its fragment generated during enzymatic digestion with trypsin (grey) as a function of incubation time. The relative amounts of the intact peptide/peptidemimetic and digestion product were calculated based on the areas of the corresponding chromatographic peaks and expressed as a percentage of the total peak area, including both the parent peptide/peptidemimetic and its fragment.

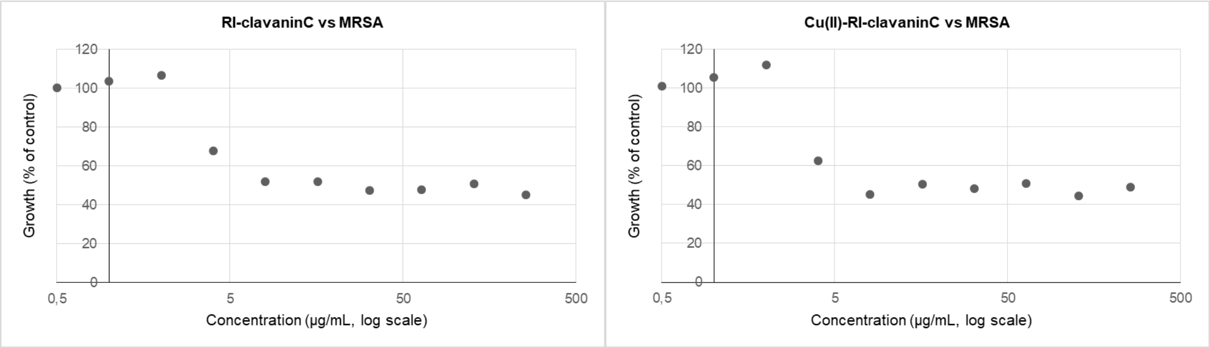

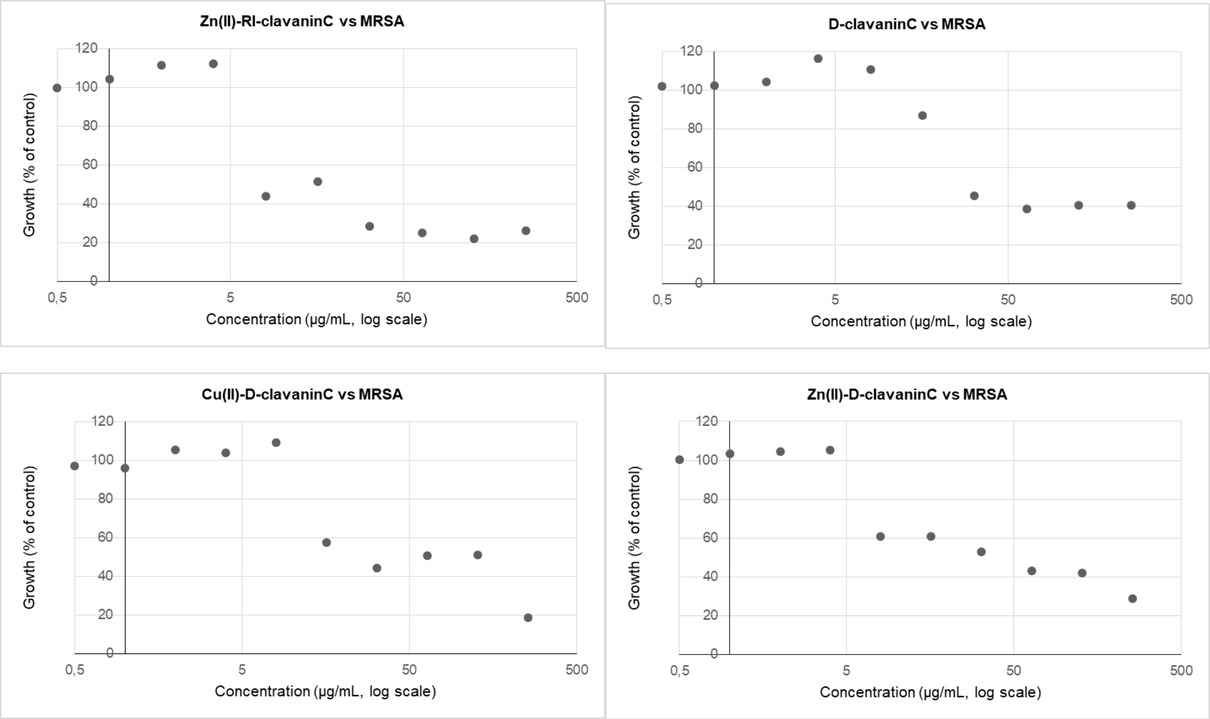

Figure S188. Dose–response curves for antimicrobial activity of clavanin C analogues and their Cu(II) and Zn(II) complexes against Staphylococcus aureus MRSA (ATCC 43300). Bacterial growth was determined spectrophotometrically (OD_{580}) after 24 h incubation and expressed as percentage relative to untreated control. Concentration-dependent inhibition of bacterial growth was observed for selected compounds.

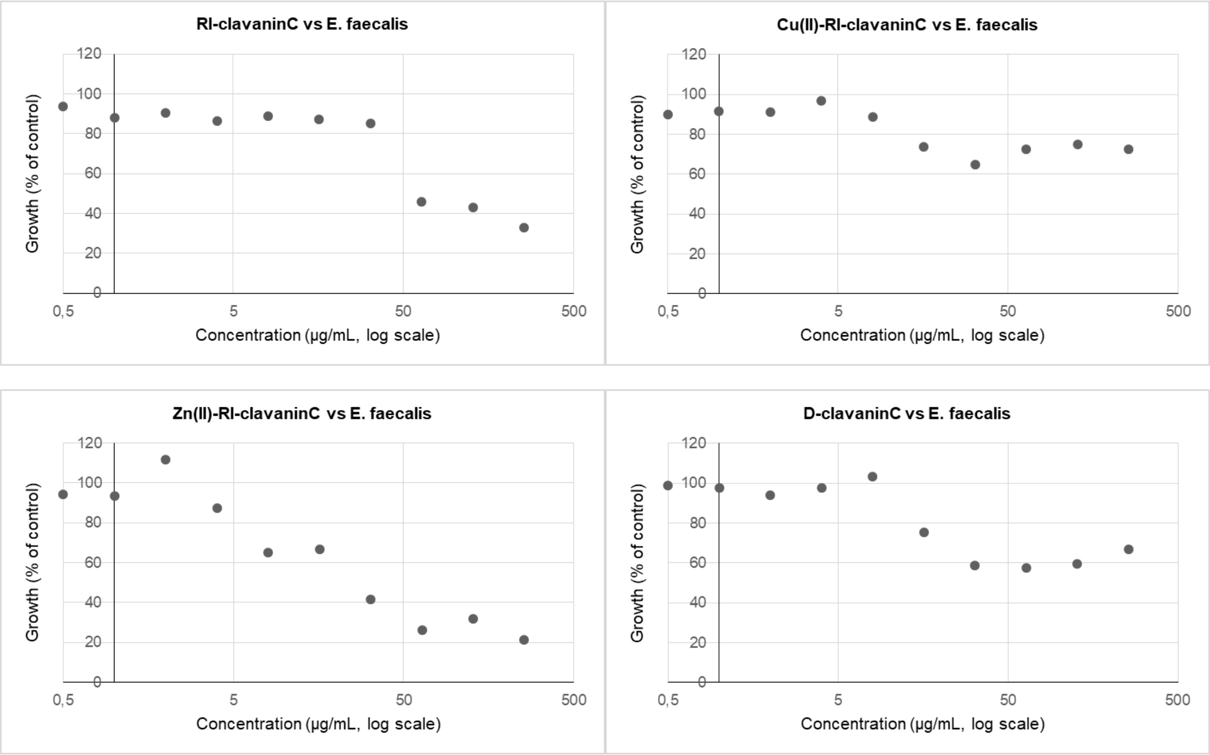

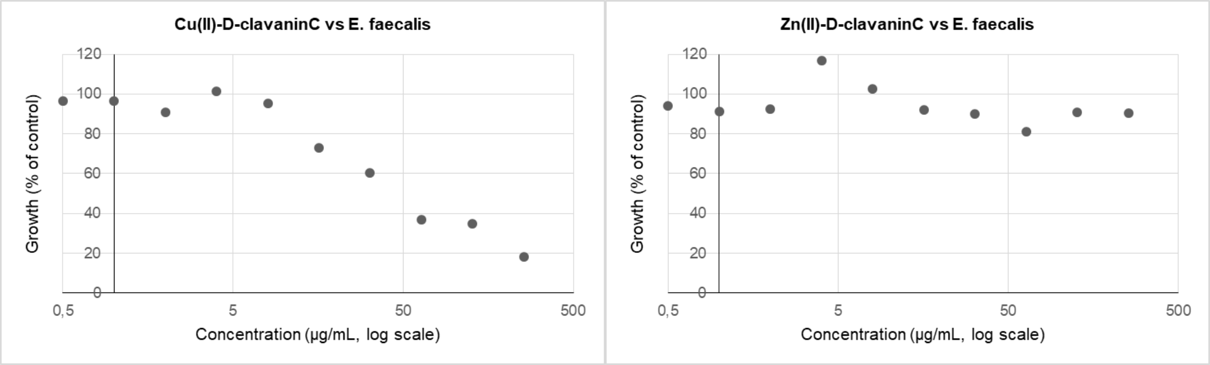

Figure S199. Dose–response curves for antimicrobial activity of clavanin C analogues and their Cu(II) and Zn(II) complexes against Enterococcus faecalis MRSA (ATCC 29212). Bacterial growth was determined spectrophotometrically (OD_{580}) after 24 h incubation and expressed as percentage relative to untreated control. Concentration-dependent inhibition of bacterial growth was observed for selected compounds.

Table S1. Examples of MIC breakpoints values from EUCAST Version 14.0 [1] for bacteria.

Enterobacterales **(for *E. coli*)**	MIC breakpoints (µg/mL)		***Enterococcus* spp. (for *E. faecalis*)**	MIC breakpoints (µg/mL)		***Staphylococcus* spp. (for *S. aureus*)**	MIC breakpoints (µg/mL)	
	S≤	R>		S≤	R>		S≤	R>
**Amoxicillin-clavulanic acid**	8	8	**Ampicillin**	4	8	**Amikacin**	8	8
**Piperacillin**	8	8	**Ampicillin-sulbactam**	4	8	**Chloramphenicol**	8	8
**Piperacillin-tazobactam**	8	8	**Amoxicillin**	4	8	**Fosfomycin iv**	32	32
**Ticarcillin**	8	16	**Amoxicillin-clavulanic acid**	4	8	**Nitrofurantoin**	64	64
**Ticarcillin-clavulanic acid**	8	16	**Nitrofurantoin**	64	64			
**Cefadroxil**	16	16						
**Cefalexin**	16	16						
**Fosfomycin iv**	32	32						
**Fosfomycin oral**	32	32						
**Nitrofurantoin**	64	64						
**Nitroxoline**	16	16						

[1] The European Committee on Antimicrobial Susceptibility Testing, Breakpoint tables for interpretation of MICs and zone diameters, 14th ed., 2024. https://www.eucast.org/clinical_breakpoints/.

Table S2. In vitro antibacterial activity of Zn(II) and Cu(II) chlorides, determined as a minimal inhibitory concentration required to inhibit the growth of 50% microorganisms (MIC50) (µg/mL); n/d, not determined. % of viability were obtained after 24h of experiment (taken from [38]).

Strain (ATCC)		% of viability in max. concentration [ug/ml]	% of viability in min. concentration [ug/ml]	MIC [ug/ml]	MBC/MFC [ug/ml]
A. baumannii	Cu(II)	97	95	512	1024
	Zn(II)	96	100	n/d	n/d
E. coli	Cu(II)	94	95	1024	n/d
	Zn(II)	92	96	1024	n/d
E. faecalis	Cu(II)	82	92	1024	n/d
	Zn(II)	76	90	n/d	n/d
P. aeruginosa	Cu(II)	77	95	512	1024
	Zn(II)	80	92	1024	n/d
S. aureus MRSA	Cu(II)	87	92	1024	n/d
	Zn(II)	86	97	1024	n/d
K. pneumoniae	Cu(II)	77	95	512	n/d
	Zn(II)	80	100	1024	n/d
C. albicans	Cu(II)	90	86	n/d	n/d
	Zn(II)	80	92	n/d	n/d
